# Water: new aspect of hydrogen bonding in the solid state

**DOI:** 10.1107/S2052252522006728

**Published:** 2022-08-05

**Authors:** Milan R. Milovanović, Ivana M. Stanković, Jelena M. Živković, Dragan B. Ninković, Michael B. Hall, Snežana D. Zarić

**Affiliations:** a Innovation Center of the Faculty of Chemistry, Studentski trg 12-16, Belgrade 11000, Serbia; b Institute of Chemistry, Technology and Metallurgy, University of Belgrade, Njegoševa 12, Belgrade, 11000 Serbia; c Department of Chemistry, Texas A&M University, College Station, TX 77843-3255, USA; d Faculty of Chemistry, University of Belgrade, Studentski trg 12-16, Belgrade 11000, Serbia; University of Iowa, USA

**Keywords:** water, hydrogen bonds, antiparallel interactions, *ab initio* calculations

## Abstract

All water–water contacts in the crystal structures from the Cambridge Structural Database with *d*_OO_ ≤ 4.0 Å have been found. These contacts were analysed on the basis of their geometries and interaction energies from CCSD(T)/CBS calculations. The results show 6729 attractive water–water contacts, of which 4717 are classical hydrogen bonds (*d*_OH_ ≤ 3.0 Å and α ≥ 120°) with most being stronger than −3.3 kcal mol^−1^. Beyond the region of these hydrogen bonds, there is a large number of attractive interactions (2062). The majority are antiparallel dipolar interactions, where the O—H bonds of two water molecules lying in parallel planes are oriented antiparallel to each other. Developing geometric criteria for these antiparallel dipoles (β_1_, β_2_ ≥ 160°, 80 ≤ α ≤ 140° and *T*_HOHO_ > 40°) yielded 1282 attractive contacts. The interaction energies of these antiparallel oriented water molecules are up to −4.7 kcal mol^−1^, while most of the contacts have interaction energies in the range −0.9 to −2.1 kcal mol^−1^. This study suggests that the geometric criteria for defining attractive water–water interactions should be broader than the classical hydrogen-bonding criteria, a change that may reveal undiscovered and unappreciated interactions controlling molecular structure and chemistry.

## Abstract 2

The geometric criteria for defining attractive water–water interactions should be broader than the classical hydrogen-bonding criteria given that attractive antiparallel interactions also exist.

## 1. Introduction

Water is omnipresent in nature. It plays significant roles in nearly all life processes: transportation of protons (Mondal *et al.*, 2020 ▸; Winkler *et al.*, 2011 ▸; Kornyshev *et al.*, 2003 ▸), protein solvation (Tompa *et al.*, 2015 ▸; Privalov & Crane-Robinson, 2017 ▸), support of polar reactions (Yau & Croft, 2013 ▸) and hydration of ions (Cooper *et al.*, 2013 ▸; Heiles *et al.*, 2015 ▸). Water also plays an important role in drug binding to proteins (Amadasi *et al.*, 2008 ▸; Spyrakis *et al.*, 2017 ▸; Luccarelli *et al.*, 2010 ▸; Samways *et al.*, 2021 ▸) and in the properties of drugs (Reddy, 2019 ▸). It is clear that life on Earth depends on its anomalous properties derived from its unique structure: small size and high polarity (Lynden Bell *et al.*, 2010 ▸; Helms, 2007 ▸) as well as flexibility (Milovanović *et al.*, 2020 ▸; Chandler *et al.*, 2015 ▸). Recent work has shown that water in confinement exhibits properties significantly different from bulk water due to frustration in the hydrogen-bonded network (Rieth *et al.*, 2019 ▸).

A fundamental ability of water is hydrogen bonding, as water forms strong hydrogen bonds to other polar molecules and itself. As defined by IUPAC, the hydrogen bond ‘is an attractive interaction’ usually presented as *X*—H⋯*Y*—*Z*, where the electropositive hydrogen atom is located between two electronegative species *X* and *Y* (Arunan *et al.*, 2011 ▸). The stability of hydrogen bonds varies in the range from −0.2 to −40 kcal mol^−1^ depending on the nature of the *X* and *Y* species and the geometry of the hydrogen bond (Steiner, 2002 ▸).

Numerous studies have been devoted to the water molecule dimer (Khaliullin *et al.*, 2009 ▸; Hoja *et al.*, 2014 ▸; Klopper *et al.*, 2000 ▸; Shank *et al.*, 2009 ▸; Rocher-Casterline *et al.*, 2011 ▸; Andrić *et al.*, 2016 ▸; Dyke *et al.*, 1977 ▸; Odutola & Dyke, 1980 ▸; Smith *et al.*, 1990 ▸; van Duijneveldt-van de Rijdt *et al.*, 2003 ▸; Tschumper *et al.*, 2002 ▸; Reinhardt & Piquemal, 2009 ▸; Engdahl & Nelander, 1987 ▸; Pardo *et al.*, 2014 ▸; Oxtoby *et al.*, 2005 ▸; Mukhopadhyay *et al.*, 2015 ▸, 2018 ▸). Most of the high-level *ab initio* calculated values for the interaction energy are in agreement with the experimental data. The calculated equilibrium interaction energy (*D*_e_) of the hydrogen-bonded water dimer, by the CCSD(T) method, is −5.02 ± 0.05 kcal mol^−1^ (Klopper *et al.*, 2000 ▸). An accurate *ab initio* and ‘hybrid’ potential energy surface gave *D*_e_ = −4.98 mol^−1^ for a water dimer. After zero-point energy correction, this potential gave a theoretical value for the dissociation energy (*D*_o_) of 3.154 ± 0.011 kcal mol^−1^ (Shank *et al.*, 2009 ▸), which is in excellent agreement with the reported spectroscopic value of 3.159 ± 0.028 kcal mol^−1^ (Rocher-Casterline *et al.*, 2011 ▸).

**Figure 1:**
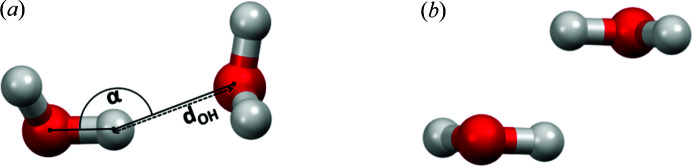
(*a*) Angle α and the distance *d*_OH_ between two water molecules; (*b*) example of an antiparallel water–water interaction.

In order to recognize hydrogen bonds in crystal structures, one has to define geometrical criteria. These criteria can then be used to extract a dataset of possible hydrogen bonds from the Cambridge Structural Database (CSD) for further analysis. A previous analysis of the number of hydrogen-bond interactions of water molecules in crystal structures showed that they start to accumulate when the *X*—H⋯*Y* (α) angle (Fig. 1 ▸) is larger than 120° (Wood *et al.*, 2009 ▸). The energy of the hydrogen bond depends on the values of the geometrical parameters, angle (α) and bond distance (*d*_OH_). The positions of hydrogen atoms in the crystal structures are important for the analysis of hydrogen bonds. Crystal structures recorded at lower temperatures have the advantage of better diffraction data at higher resolution. Positions of hydrogen atoms can be determined from difference Fourier maps (Blake *et al.*, 2009 ▸). However, in this way the *X*—H bond lengths determined are usually shorter that the true *X*—H bond lengths obtained from neutron diffraction. The *X*—H bond lengths obtained by neutron diffraction are over 0.1 Å longer. Normalization is used to correct *X*—H bond lengths (Allen & Bruno, 2010 ▸).

Hydrogen bonds are generally considered strong when the H⋯*Y* distance is 2.2 to 2.5 Å and the *X*—H⋯*Y* angle is 170 to 180°, whereas for weak hydrogen-bond interactions, the H⋯*Y* distance is larger than 3.2 Å and the bond angle is less than 130° (Steiner, 2002 ▸). Between strong and weak interactions are the moderate-strength interactions with H⋯*Y* distances between 2.5 and 3.2 Å and *X*—H⋯*Y* angles between 130 and 170° (Steiner, 2002 ▸).

The calculated interaction energies of ten water dimer geometries (Smith *et al.*, 1990 ▸; van Duijneveldt-van de Rijdt *et al.*, 2003 ▸; Tschumper *et al.*, 2002 ▸; Reinhardt & Piquemal, 2009 ▸) show that, even when the geometries do not satisfy the usual geometric criteria for hydrogen bonding (Steiner, 2002 ▸; Wood *et al.*, 2009 ▸), the interactions can be attractive. This indicates that the geometric area with attractive interactions between water molecules is much broader than previously realized. Although calculations have shown that these unusual attractions between water molecules exist, the role of these interactions has been neglected.

Water molecules also contribute to a variety of other interactions in unexpected ways (Janjić *et al.*, 2011 ▸, 2014 ▸; Ninković *et al.*, 2012 ▸; Ostojić *et al.*, 2008 ▸). For example, the interaction energy between water molecules having one O—H bond parallel to the aromatic ring of a benzene molecule is −2.45 kcal mol^−1^ (Janjić *et al.*, 2011 ▸). Furthermore, the hydrogen bonding of water molecules to pyridine can strengthen the stacking interaction, since in the structure of the stacked pyridine–water dimers, the contribution of the local parallel oriented interactions of the water molecules with pyridines is −2.98 kcal mol^−1^ (Ninković *et al.*, 2012 ▸).

Based on these unexpected interactions, we wondered if water–water interactions could display a wider variety of attractive interactions beyond typical hydrogen bonds. In this work, we analysed geometries for all water–water interactions in the CSD and calculated their interaction energies at the accurate CCSD(T)/CBS level. Based on these data, our results indicate two types of attractive water–water interactions; the first type involves the classical hydrogen bond, whereas the second type involves antiparallel O—H bond interactions (Fig. 1 ▸). To the best of our knowledge, this is the first study that provides very accurate interaction energies of all relevant water–water interactions from the CSD, and examines the geometrical criteria for recognizing attractive water–water interactions.

## 2. Results and discussion

**Figure 2:**
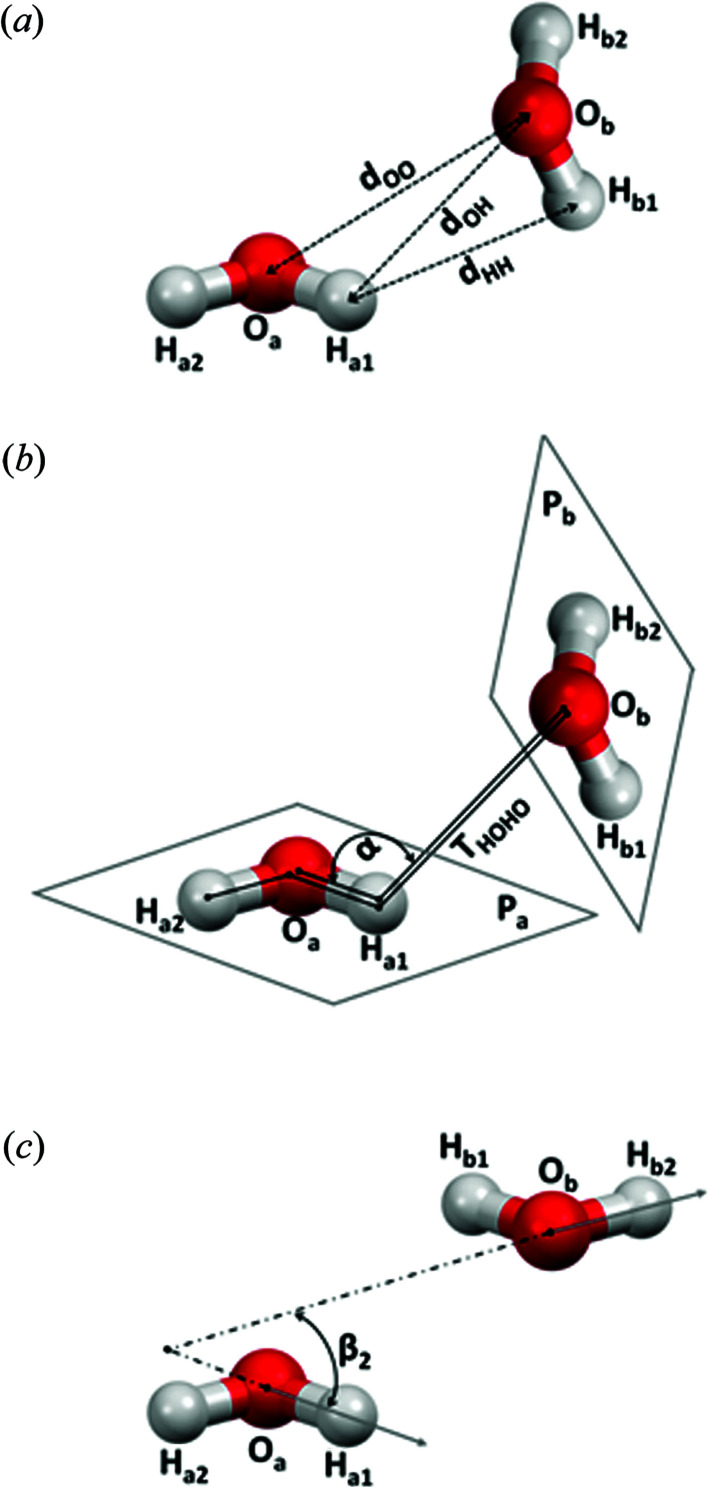
Geometric parameters and atom labelling used for the description of intermolecular interactions between two water molecules, a and b. (*a*) The distance between two oxygen atoms is *d*_OO_. H_a1_ represents the hydrogen atom that has the shortest non-bonded O⋯H distance (O_b_⋯H_a1_), which is *d*_OH_. H_b1_ represents the hydrogen atom that has a shorter H_a1_⋯H_b_ distance, which is *d*_HH_. (*b*) The angle Oa—H_a1_⋯O_b_ is α. *P*_a_/*P*_b_ is the dihedral angle between two water molecule planes. The torsion angle H_a2_—O_a_—H_a1_—O_b_ is *T*_HOHO_. (*c*) Angles between vectors containing O—H bonds are denoted β_*n*_. β_1_ represents the angle O_a_—H_a1_⋯O_b_—H_b1_, β_2_ represents the angle O_a_—H_a1_⋯O_b_—H_b2_. Only β_2_ is shown, the others have been omitted for clarity.

Applying the criteria detailed in the Methodology[Sec sec4] to the CSD search produced 9928 water–water contacts with a distance between two oxygen atoms (*d*_OO_) shorter than 4.0 Å (Fig. 2 ▸). For all of the above interactions we calculated interaction energies at the accurate CCSD(T)/CBS level. To avoid uncertainties of the calculated interaction energies, contacts with interaction energies in the range −0.3 to +0.3 kcal mol^−1^ were excluded (583 contacts). The calculated interaction energies revealed 6729 (72%) attractive (with energy less than −0.3 kcal mol^−1^) and 2616 (28%) repulsive (with energy greater than 0.3 kcal mol^−1^) water–water contacts (Fig. S1 of the supporting information).

**Figure 3:**
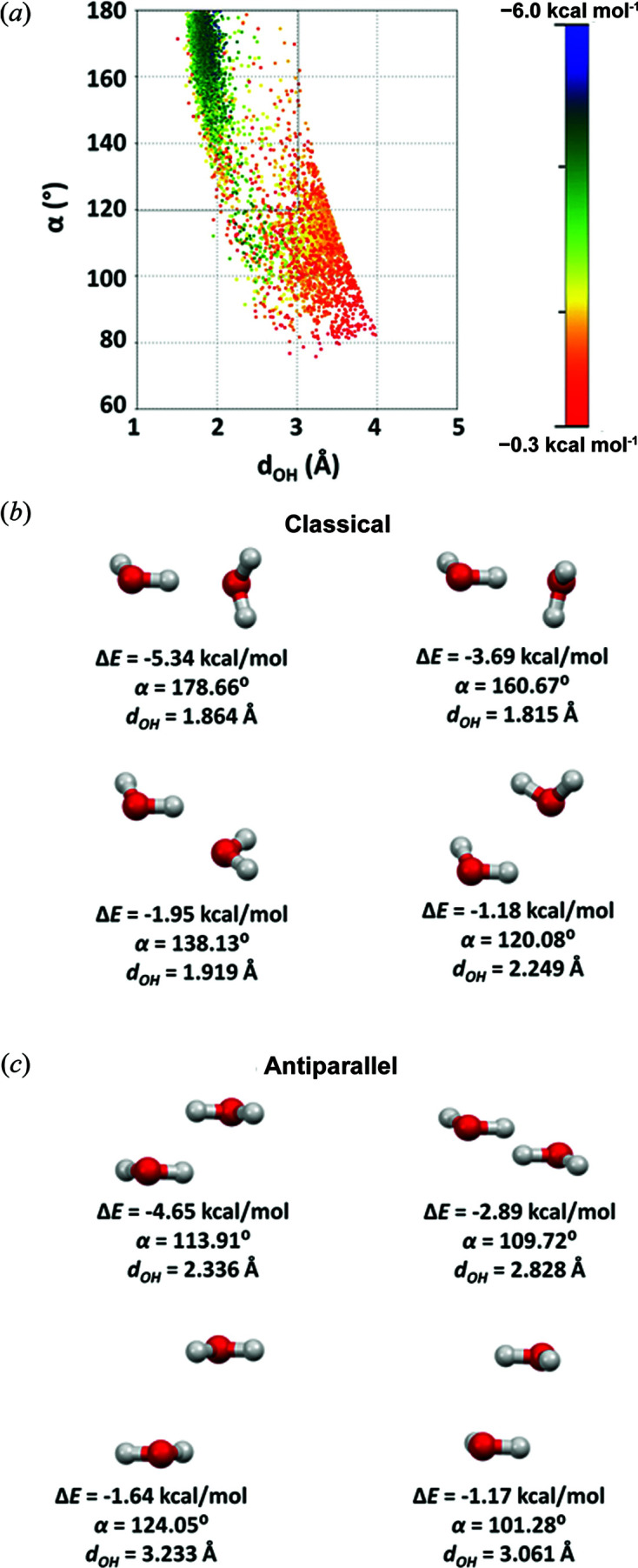
Attractive water–water contacts in the CSD structures. (*a*) Plot of the distance *d*_OH_ versus angle α. The colours correspond to CCSD(T)/CBS interaction energies, as shown in the scale. Graphical representations and CCSD(T)/CBS interaction energies of some examples of water–water interactions from the CSD, with (*b*) classical hydrogen bonds and (*c*) antiparallel interactions.

The plot of the distance *d*_OH_ and the angle α of attractive interactions [Fig. 3 ▸(*a*)] indicates two regions: one that corresponds to classical hydrogen bonds [*d*_OH_ < 3.0 Å and α > 120° (Steiner, 2002 ▸; Wood *et al.*, 2009 ▸)], where we can notice clustering of relatively strong interactions (blue and green dots) that show shortening of *d*_OH_ distances with increasing α angles. Structural examples of these classical hydrogen bonds [Fig. 3 ▸(*b*)] appear strongest when α is close to 180°, as anticipated, and the weaker with smaller α angles. In addition, there are many attractive interactions that do not show this dependence; the majority of these interactions are in the region α < 120°.

Detailed visual analysis of these interactions shows that, in most of the stronger interactions, the two water molecules lie in nearly parallel planes. In these contacts, the attractive interactions arise from two antiparallel O—H dipoles. Examples of these water–water interactions are shown in Fig. 3 ▸(*c*). The calculated energies for these antiparallel interactions [Fig. 3 ▸(*c*)] indicate the importance of these interactions, as some have energies similar to classical hydrogen bonds, some even as large as −4.65 kcal mol^−1^. The representative geometries of these antiparallel interactions shown in Fig. 3 ▸(*c*) can help in their recognition in crystal structures.

To find the geometric criteria for these two types of attractive water–water interactions, we analysed the interaction energies (Δ*E*), the distance *d*_OH_, the angle α, the torsion angle *T* and angles between vectors [β_1_ O_a_—H_a1_⋯O_b_—H_b1_ and β_2_ O_a_—H_a1_⋯O_b_—H_b2_ (Fig. 2 ▸)]. Two other possible angles between vectors β_3_ (O_a_—H_a2_⋯O_b_—H_b1_) and β_4_ (O_a_—H_a2_⋯O_b_—H_b2_) were not considered since we found that β_1_ and β_2_ are sufficient for the purposes of this classification.

**Figure 4:**
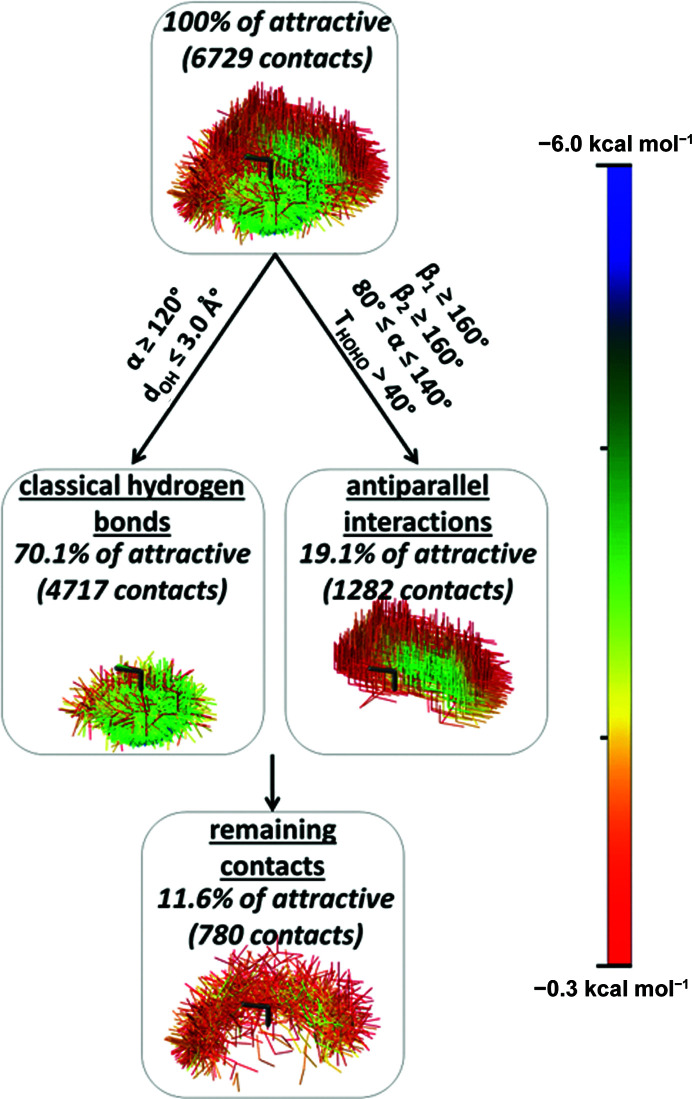
Sets of attractive water–water contacts found in the CSD. In the graphical representations of water–water contacts, one water molecule was positioned in the centre (shown in dark grey). The other water molecules from every water–water contact are shown in the colour representing the CCSD(T)/CBS interaction energies, as shown in the scale.

The geometric criteria to separate these two main groups of attractive interactions are presented in Fig. 4 ▸. By applying criteria for hydrogen bonds *d*_OH_ ≤ 3.0 Å and α ≥ 120°, a subset containing 4717 attractive contacts (*ca* 70.1% of all attractive contacts) is obtained (Figs. 4 ▸, S2 and S3). This subset can be labelled as a group of classical hydrogen-bonded water molecules.

Detailed visual analysis on the attractive interactions that are not classical hydrogen bonds shows many contacts that have parallel orientations of water planes as well as antiparallel O—H bonds, as mentioned above.

We found geometric criteria to describe the majority of these water–water attractive interactions with antiparallel O—H bonds: β_1_, β_2_ ≥ 160°, 80 ≤ α ≤ 140° and *T*_HOHO_ > 40°. Namely, the crucial criterion for antiparallel dipolar interaction is the angle between the dipoles (*i.e.* vectors containing O—H bonds). Therefore, angle β (Fig. 2 ▸) should be close to 180° [*i.e.* one of the vector angles (β_1_ or β_2_) should be ≥160°]. In order to exclude the most repulsive antiparallel interactions, we found that the torsion angle *T*_HOHO_ is important and should be >40°. Hence, after applying the first criterion: β_1_, β_2_ ≥ 160° on all considered contacts, most contacts with *T*_HOHO_ < 40° are repulsive (Fig. S4). A third criterion requires that the hydrogen-bonding angle α (Fig. 2 ▸) should be in the range 80–140°. Namely, after applying the criteria β_1_, β_2_ ≥ 160 and *T*_HOHO_ > 40°, most contacts with α < 80° and α > 140° are repulsive (Fig. S5).

Applying these criteria for antiparallel interactions produced a set of 1282 contacts (*ca* 19.1% of all attractive contacts; Figs. 4 ▸, S6 and S7). We also performed SAPT analysis on some geometries belonging to this set of antiparallel interactions. The results of the SAPT analysis (Tables 1 ▸ and S1 of the supporting information) show that the main attractive forces of these antiparallel interactions are electrostatic in nature, which arise from the interaction of antiparallel O—H bonds (*i.e.* local dipoles).

There is a small overlap between the two sets with attractive water–water interactions. Namely, the set of classical hydrogen-bound water molecules described above (*d*_OH_ ≤ 3.0 Å and α ≥ 120°) also contains 51 antiparallel interactions (*i.e. ca* 0.8% of all attractive water–water contacts; Fig. S8).

Although hydrogen bonds and antiparallel interactions are the two most important sets of attractive interactions, there are some attractive interactions in the remaining set, *i.e.* 780 attractive contacts (*ca* 11.6% of the total attractive contacts; Fig. 4 ▸, S9 and S10).

**Figure 5:**
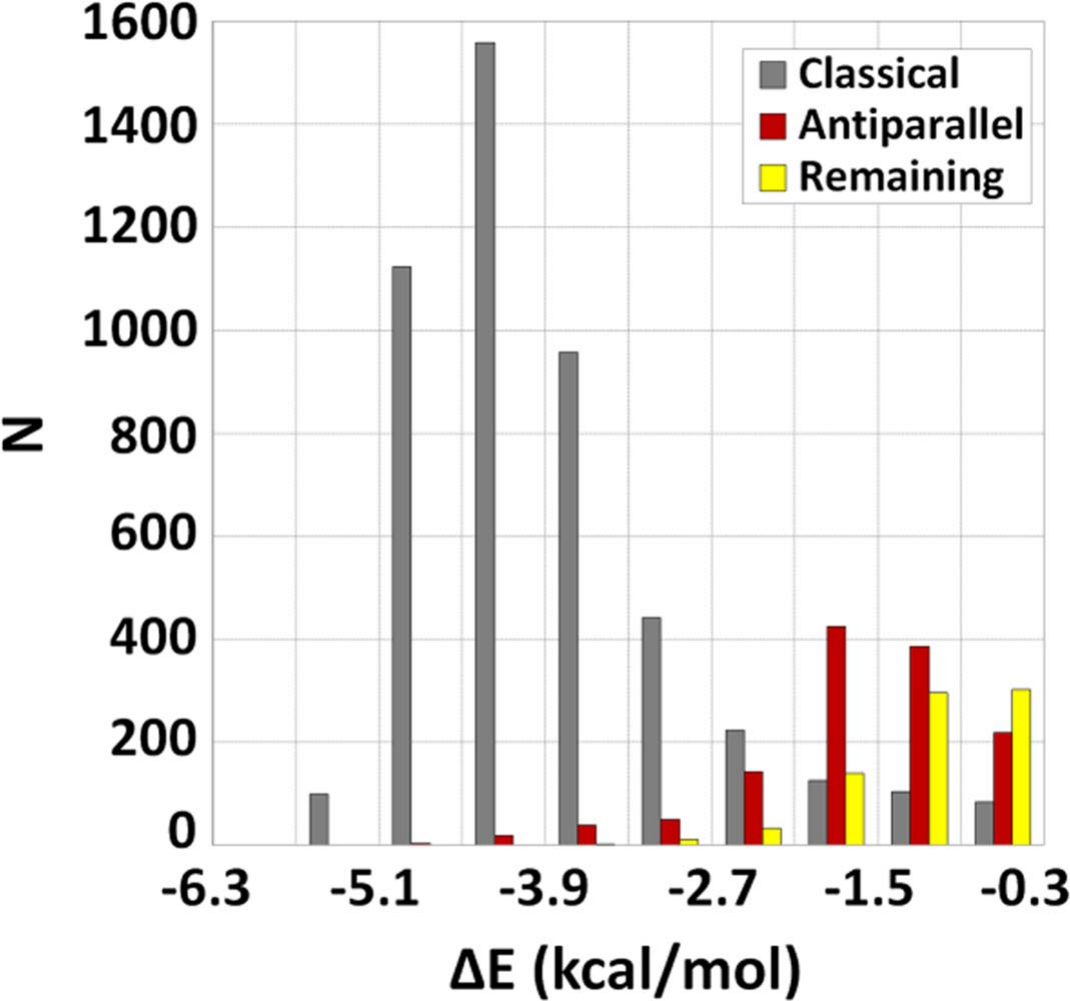
Distribution of interaction energies of attractive water–water contacts found in the CSD after applying the criteria for classical hydrogen bonds (*d*_OH_ ≤ 3.0 Å and α ≥ 120°) and antiparallel interactions (β_1_, β_2_ ≥ 160°, 80 ≤ α ≤ 140° and *T*_HOHO_ > 40°), calculated at the CCSD(T)/CBS level of theory.

Fig. 5 ▸ presents the interaction energies for the three sets of attractive interactions: classical, antiparallel and remaining. The data show that the classical hydrogen-bonded water molecules are the strongest; most are stronger than −3.5 kcal mol^−1^. Hence, the classical ones are the most important because of their large number and strength. The interaction energies of the antiparallel interactions are up to −4.7 kcal mol^−1^, whereas most of the contacts have interaction energies in the range −0.9 to −2.1 kcal mol^−1^. Although these interactions are significantly weaker, their energies still can be important in competition with other weak noncovalent interactions in crystal structures and other molecular systems (Politzer & Murray, 2020 ▸; Pradeepa & Dasb, 2013 ▸; Mahmudov *et al.*, 2017 ▸; Ghosh *et al.*, 2019 ▸; Thanasekaran *et al.*, 2021 ▸).

The attractive interactions in the remaining set are mostly less than *ca* −1.5 kcal mol^−1^. Since these interactions are quite weak in comparison with hydrogen-bonded and antiparallel water–water interactions, they are less important.

Analyses of other geometric parameters show that the contacts of the classical hydrogen bound water molecules have the *P*_a_/*P*_b_ angle (the angle between their two molecular planes, Fig. 2 ▸) in the whole range (0–90°) but with a tendency towards 90°, whereas the *P*_a_/*P*_b_ angle in the set of interactions with antiparallel O—H bonds is almost exclusively 0° (Fig. S11). The vector angles β_1_ and β_2_ (Fig. 2 ▸ and S11) in most of the classical hydrogen-bound water molecules span in the approximate range 30–120° and *ca* 0–100°, respectively. The vector angles of antiparallel interactions of water molecules (Fig. 2 ▸ and S11) are mutually related and are in either the range 160–180° and *ca* 65–85°, respectively, or vice versa. In addition, the two groups, classical hydrogen bonds and antiparallel interactions, contain some repulsive contacts. Namely, classical hydrogen bonds contain 458 repulsive contacts (*ca* 17.5% of all repulsive contacts; Figs. S2 and S3), whereas antiparallel interactions contain only 50 repulsive contacts (1.9% of all repulsive contacts; Figs. S6 and S7).

To prove reliability of our data we performed a temperature analysis, at which the considered crystal structures were recorded; 46% of the structures used in our work were recorded at temperatures below −78°C (Fig. S29). We also carried out an analysis using only structures solved at low temperatures. In comparison with the data presented in Figs. 3 ▸–5, we obtained very similar data using only structures solved at temperatures below −78°C (Figs. S30–S33). The percentages for each group of contacts [*i.e.* classical hydrogen bonds, antiparallel interactions and remaining contacts (Fig. S31)] are similar to those presented in the Fig. 4 ▸. In addition, we performed the analysis using data from crystal structures chosen in a more restrictive way; we considered only structures in which all atoms (including hydrogen atoms) were solved by a difference Fourier map method and all hydrogen atom parameters were refined (Figs. S22, S24, S26 and S28). Again, these distributions are very similar to those presented in Figs. 3–5. These data obtained with more restrictive criteria indicate that our conclusions on the type of water–water interactions in crystal structures are reliable.

The distributions of distances *d*_OH_ and *d*_OO_ for antiparallel water–water interactions with respect to their interaction energies are shown in Fig. S12. There is a general trend that, with an increase in distances *d*_OH_ and *d*_OO_, the strength of the interaction decreases, although there are weak interactions with relatively short distances.

Some examples of crystal structures containing antiparallel water–water interactions and their packing diagrams are given in Figs. S13–S19. Note that an antiparallel water dimer can be found: (*a*) alone in the core (Figs. S13–S14) or on the side (Figs. S15–S16) of the crystallographic cell, (*b*) in shorter (Figs. S17–S18) or longer (Figs. S19) chains of water molecules. The packing diagrams indicate that two water molecules forming an antiparallel interaction can form additional interactions with the surrounding water molecules. These additional interactions contribute to the stabilization of supramolecular structures in crystals. It seems that, besides a stabilization role, the water dimer with the antiparallel interaction, owing to its symmetry, has a role in crystal packing. Interaction energies and geometric parameters of water–water contacts in the selected CSD crystal structures (Figs. S13–S19) are given in Table S3. In structures with water chains there are antiparallel interactions and classical hydrogen bonds between water molecules. In all cases each water molecule involved in the antiparallel interaction forms a classical hydrogen bond with other water molecules. In some crystal structures (MIKWIP and AQOXCU) antiparallel water–water interactions are stronger (−3.61 and −4.15 kcal mol^−1^, respectively) than classical hydrogen bonds (−2.09 and −2.93 kcal mol^−1^, respectively).

As a supplement to the study of the interaction of two water molecules, we performed a similar analysis on the interactions between other molecules containing an O—H bond. A great majority of these structures (*ca* 90.4%) are alcohols. The results showed that 58.9% of all potentially attractive interactions could be labelled as classical hydrogen bonds, while *ca* 12.4% could be classified as antiparallel interactions, containing two antiparallel O—H bonds (Fig. S20). Like antiparallel water–water interactions, there are clear preferences of the dihedral angle (*P*_a_/*P*_b_) near 0° and the vector angle (β) near 180° [Fig. S20(*b*)] in cases of molecular fragments with antiparallel O—H bonds. Dipole interactions between antiparallel polar bonds are important interactions, not only between water and alcohol molecules, but in many other molecules. For example, the interaction of antiparallel dipoles is important in benzene–benzene stacking at large offsets, where two antiparallel C—H bonds interact (Ninković *et al.*, 2020 ▸). Similar interactions are observed in benzene–water stacking, where C—H and O—H bonds are in antiparallel orientations (Janjić *et al.*, 2011 ▸).

In addition, we can speculate that one of the possible reasons for the appearance of the antiparallel water–water interaction lies in its symmetry. Namely, by looking into crystal structures containing strong antiparallel interactions, we noticed these appear when a particular symmetry of solvent molecules is present (for example, structures with a centre of inversion).

A free web server (http://www.chem.bg.ac.rs/~szaric/water_interactions/water_interactions.py) was made for determining the water–water interaction type: either classical hydrogen bonding and antiparallel interaction. A cif and the desired atom names of the two interacting water molecules should be given as the input. As an output the program provides the interaction type and Cartesian coordinates of the two water molecules labelled by the criteria in this paper, as well as all the geometrical parameters used for determining the interaction type. For details see section S2 of the supporting information.

## 3. Conclusions

The water–water contacts in the crystal structures from the CSD with *d*_OO_ ≤ 4.0 Å were extracted and studied. Analyses of the contact geometries and calculations of accurate CCSD(T)/CBS interaction energies of all contacts were performed. The calculated energies showed 6729 attractive and 2616 repulsive contacts. The data showed that the common geometric criteria for hydrogen bonding (*d*_OH_ ≤ 3.0 Å and α ≥ 120°) cannot separate attractive and repulsive water–water interactions. Though in the region of hydrogen bonding (*d*_OH_ ≤ 3.0 Å and α ≥ 120°) there is large number of attractive contacts (4715 contacts), outside this region there is also a significant number of attractive interactions (2062 contacts). Detailed analysis indicated that a fairly important group of attractive interactions are antiparallel interactions, where O—H bonds of the two water molecules are antiparallel. By developing geometric criteria for these antiparallel interactions (β_1_, β_2_ ≥ 160°, 80 ≤ α ≤ 140° and *T*_HOHO_ > 40°), we obtained 1282 attractive contacts of this class. Although the classical hydrogen bonds are quite strong (stronger than −3.3 kcal mol^−1^), the attractive water–water interactions with antiparallel orientations of the O—H bonds can be as strong as −4.7 kcal mol^−1^ with most between −0.9 and −2.1 kcal mol^−1^.

Based on this study, we suggest that the geometric criteria for defining attractive water–water interactions should be broader than just the classical hydrogen-bonding criteria. Furthermore, other O—H bonds such as those in alcohols also show these additional important attractive interactions. In general, this expanded definition for attractive interactions to include antiparallel dipolar interactions can be applied to other molecules. Our forthcoming studies will be dedicated to detailed examination of geometrical parameters that can be used to describe interactions between molecules containing other polar bonds.

## 4. Methodology

### 4.1. Searching the Cambridge Structural Database

The statistical study was based on the data from the Cambridge Structural Database (CSD) [November 2018 released, August 2019 updated, version 5.40 (Groom *et al.*, 2016 ▸]. The CSD search was performed in order to obtain the crystal structures containing at least one non-coordinated water molecule and at least one O—H bond. The search program *ConQuest* (version 2.0.3; Bruno *et al.*, 2002 ▸) was used to retrieve crystal structures resolved by X-ray diffraction analysis satisfying the following criteria: (*a*) distance between two oxygen atoms *d*_OO_ ≤ 4.0 Å; (*b*) a crystallographic *R* factor ≤ 5%; (*c*) error-free coordinates according to the criteria used in the CSD; (*d*) O—H bond lengths normalized using the CSD default O—H bond lengths (0.993 Å), that is, in accordance with a typical O—H bond length from neutron diffraction analysis (Allen & Bruno, 2010 ▸), adjustments of O—H⋯O angles were not made; (*e*) no ionic structures; (*f*) no polymer structures; (*g*) no powder structures; (*h*) no disordered structures; and (*i*) 3D coordinates determined. An additional restriction that could minimize structures with incorrectly introduced hydrogen atoms was achieved by taking into consideration only structures with bond angles (H—O—H) in the range 96.4–112.8° (Milovanović *et al.*, 2020 ▸). Statistical analysis and quantum chemical calculations were performed on the structures satisfying all the above-mentioned criteria.

Statistical analysis of crystal structures resolved by neutron diffraction analysis was not performed due to an extremely small number of available structures, we found only eight water–water contacts in these structures. General reliability of hydrogen atom positions in X-ray solved structures was shown previously (Ostojić *et al.*, 2008 ▸). Namely, detailed analyses show accordance of the data obtained from the CSD and data obtained by water hydrogen atoms located from the difference Fourier maps.

In order to validate the reliability of the results, the energy calculations and geometric analysis were performed on structures in which all atoms (including hydrogen atoms) were solved by the difference Fourier map method and all hydrogen atom parameters were refined. In addition, we examined temperatures the considered crystal structures were recorded at and we performed the same type of analysis for the subset of structures recorded at ≤−78°C.

The geometric parameters used to describe and analyse water–water interactions are given in Fig. 2 ▸.

### 4.2. Computational methods

To obtain interaction energies of two interacting water molecules, *ab initio* calculations were performed using the *Gaussian09* program package (Frisch *et al.*, 2016 ▸). The interaction energies of all water–water geometries found in the CSD satisfying the criteria described in the CSD methodology[Sec sec4.1] (9928 water dimers) were calculated at a very accurate level of theory, *i.e.* at CCSD(T)/CBS (Cížek, 1969 ▸; Purvis & Bartlett, 1982 ▸; Scuseria *et al.*, 1988 ▸; Scuseria & Schaefer, 1989 ▸; Pople *et al.*, 1987 ▸) level [the so called gold standard, Mackie and DiLabio method (Mackie & DiLabio, 2011 ▸)]. The Gaussian input file for the whole set of interactions was written with python (https://www.python.org). To provide insight into the nature of the interactions an SAPT analysis (Jeziorski *et al.*, 1994 ▸) was performed with the *PSI4* program (Turney *et al.*, 2012 ▸). We used an SAPT method with a density-fitting approximation (DF-SAPT2+3) (Hohenstein & Sherrill, 2010 ▸) and the *def2-qzvppd* basis set, since using this basis set gave results in good agreement with the accurate CCSD(T)/CBS energies (Tables 1 and S1). The coordinates for visualization were obtained by a script prepared in python. Finally, the visualization of the water–water contacts was carried out using the *VMD* (Humphrey *et al.*, 1996 ▸) or *Mercury* software (version 4.2.0; Macrae *et al.*, 2008 ▸).

## 5. Related literature

The following references are cited in the supporting information: Al-Harthy *et al.* (2019 ▸); Cui *et al.* (2012 ▸); Gao *et al.* (2008 ▸); Gavette *et al.* (2011 ▸); Ghosh *et al.* (2017 ▸); Ghosh *et al.* (2010 ▸); Gilardi & Evans (2003 ▸); Golovnev *et al.* (2017 ▸); Gudasi *et al.* (2005 ▸); Hu *et al.* (2003 ▸); Jiao *et al.* (2016 ▸); Karipides (1981 ▸); Keana *et al.* (1983 ▸); Klapötke *et al.* (1999 ▸); Korotaev *et al.* (2012 ▸); Korotaev *et al.* (2013 ▸); Lu *et al.* (2015 ▸); Ma *et al.* (2018 ▸); Maiti *et al.* (2014 ▸); Ohno *et al.* (2018 ▸); Ohui *et al.* (2019 ▸); Pajunen & Näsakkälä (1980 ▸); Rao & Rao (2007 ▸); Sen *et al.* (2006 ▸); Sun *et al.* (2005 ▸); Sun *et al.* (2006 ▸); Tao & Wang (2011 ▸); Tang *et al.* (2017 ▸); Vishweshwar *et al.* (2002 ▸); Wang & Seyedsayamdost (2017 ▸); Zheng *et al.* (2006 ▸); Zhou *et al.* (2015 ▸); Zhu *et al.* (2012 ▸).

## Supplementary Material

Supporting figures and tables. DOI: 10.1107/S2052252522006728/lq5046sup1.pdf

## Figures and Tables

**Table 1:** Results of the SAPT analysis on some of the geometries of water–water contacts belonging to the group of antiparallel interactions (β_1_, β_2_ ≥ 160°, 80° ≤ α ≤ 140° and *T*_HOHO_ > 40°)

Refcode	CCSD(T)/CBS (kcal mol^−1^)	Electrostatics (kcal mol^−1^)	Exchange (kcal mol^−1^)	Induction (kcal mol^−1^)	Dispersion (kcal mol^−1^)	Net dispersion (kcal mol^−1^)	Total SAPT2+3 (kcal mol^−1^)
IGOLEX	−4.65	−6.55	5.32	−1.19	−2.18	3.14	−4.60
QICTAY	−4.04	−8.21	8.94	−1.80	−2.92	6.02	−3.98
GITHUP	−3.52	−3.75	2.17	−0.50	−1.38	0.79	−3.46
CARNEO	−3.03	−2.95	1.44	−0.35	−1.12	0.32	−2.99
XECJEU01	−3.01	−2.63	0.58	−0.23	−0.74	−0.16	−3.02
TEQKOQ	−2.00	−1.76	0.37	−0.14	−0.47	−0.09	−1.98
HUBGAO	−2.00	−1.76	0.63	−0.19	−0.67	−0.04	−1.98
KUXTAZ	−1.98	−3.05	2.79	−0.36	−1.40	1.39	−2.03
AQMLCO	−1.72	−2.53	2.29	−0.30	−1.21	1.08	−1.75
OCIDAI	−1.00	−0.85	0.16	−0.05	−0.28	−0.12	−1.02
PASPOR	−1.00	−1.02	0.70	−0.11	−0.60	0.10	−1.03
