# Supplementary material for: Water: new aspect of hydrogen bonding in the solid state
Source: IUCrJ. 2022 Aug 5;9(Pt 5):639–47. doi: 10.1107/S2052252522006728 (PMC9438494; doi:10.1107/S2052252522006728)
Supplement: Supplementary file 1 [file m-09-00639-sup1.pdf]

# IUCrJ

**Volume 9 (2022)**



**Supporting information for article:**

## Water: New Aspect of Hydrogen Bonding in the Solid State

**Milan R. Milovanović, Ivana M. Stanković, Jelena M. Živković, Dragan B. Ninković, Michael B. Hall and Snežana D. Zarić**

## S1. Graphic representations

This section contains supporting figures and graphs mentioned in the main text of the article.

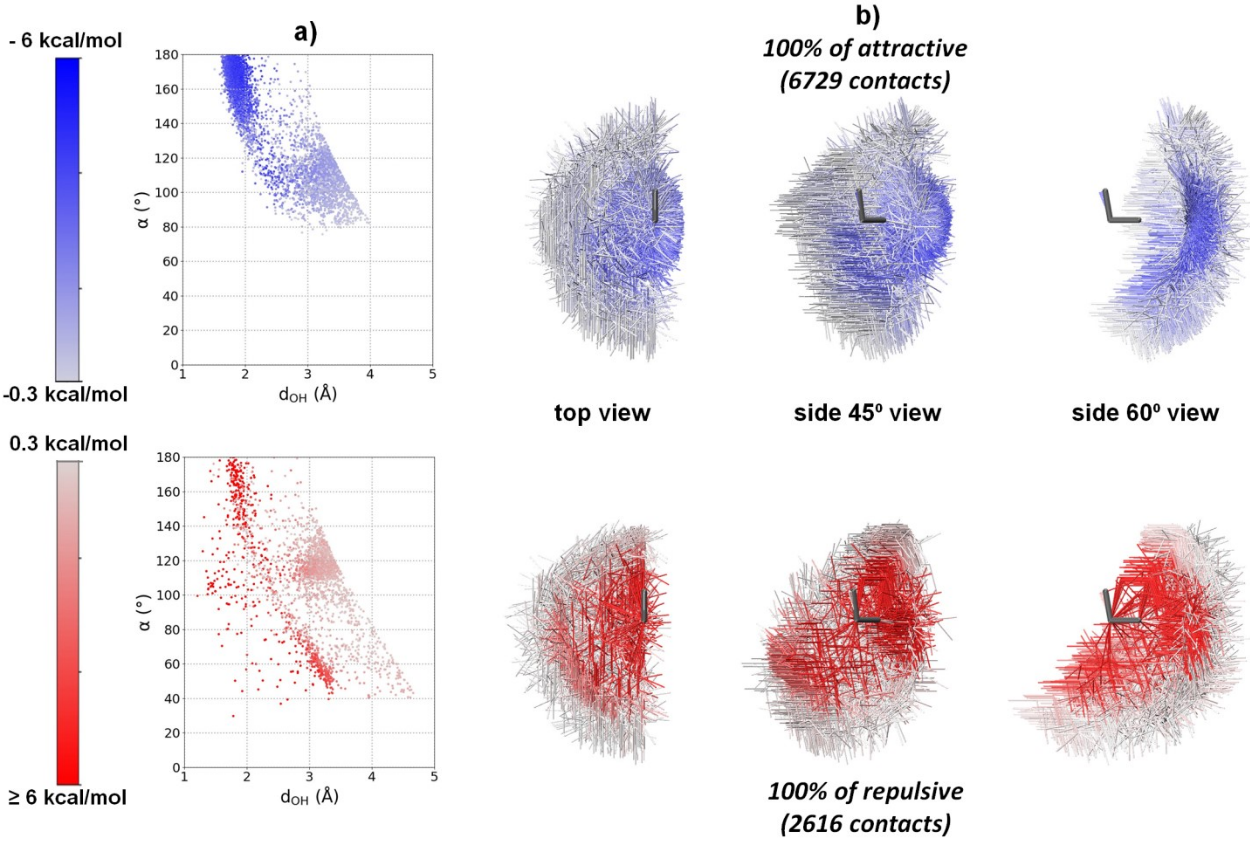


**Figure S1** Water-water contacts in the CSD structures. Colors correspond to interaction energies, blue (attractive) and red (repulsive), as was shown by the scale. *a)* The plots of the distance $d_{OH}$ (**Figure 2**) versus the angle $\alpha$ (**Figure 2**) for all attractive (6729) and all repulsive (2616) water-water contacts found in CSD. *b)* Graphical representations of water-water contacts corresponding to *a*. In the graphical representations of water-water contacts, one water molecule was positioned in the center (shown in dark gray licorice) and the other water molecule from every water-water contact is shown in the color representing the energy of the interaction.

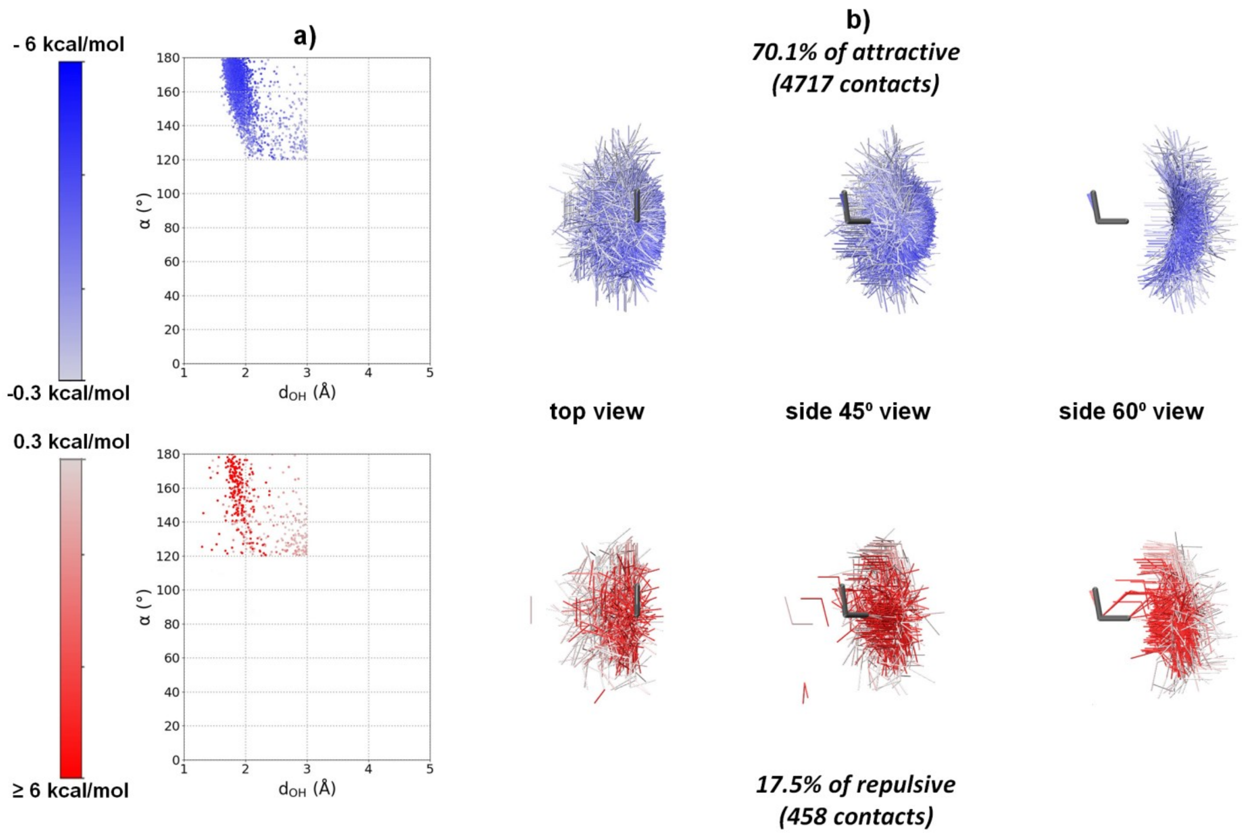


**Figure S2** Water-water contacts in the CSD structures. Colors correspond to interaction energies, blue (attractive) and red (repulsive), as was shown by the scale. *a)* The plots of the distance $\boldsymbol{d_{OH}}$ (**Figure 2**) versus the angle $\boldsymbol{\alpha}$ (**Figure 2**) for attractive (4717) and repulsive (458) contacts of classical hydrogen bonds found in CSD after applying criteria: $\boldsymbol{d_{OH}} \leq 3.0$ Å and $\boldsymbol{\alpha} \geq 120°$ (**Figure 4**). *b)* Graphical representations of water-water contacts corresponding to *a*. In the graphical representations of water-water contacts, one water molecule was positioned in the center (shown in dark gray licorice) and the other water molecule from every water-water contact is shown in the color representing the energy of the interaction.

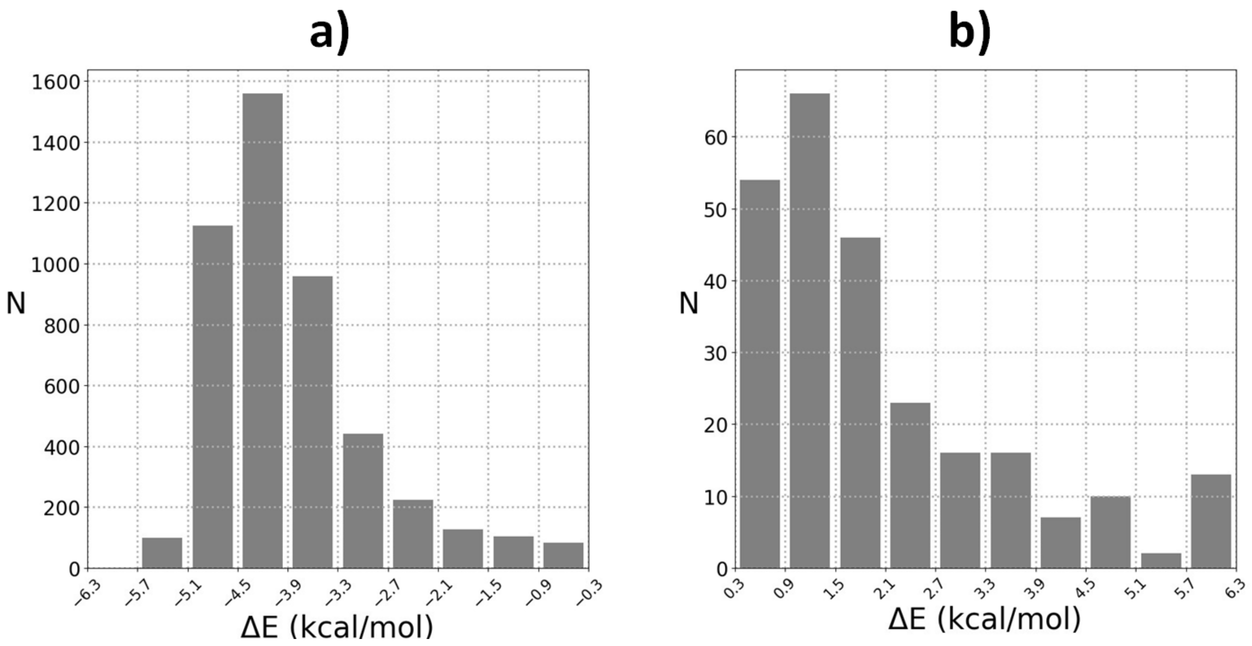


**Figure S3** The distribution of interaction energies of classical hydrogen bonds found in CSD after applying criteria: $\boldsymbol{d_{OH}} \leq 3.0$ Å and $\boldsymbol{\alpha} \geq 120°$ (**Figure 4**), *a)* 4717 attractive and *b)* 458 repulsive contacts, calculated at CCSD(T)/CBS level of theory. The distribution corresponds to the structures shown in **Figure S2**.

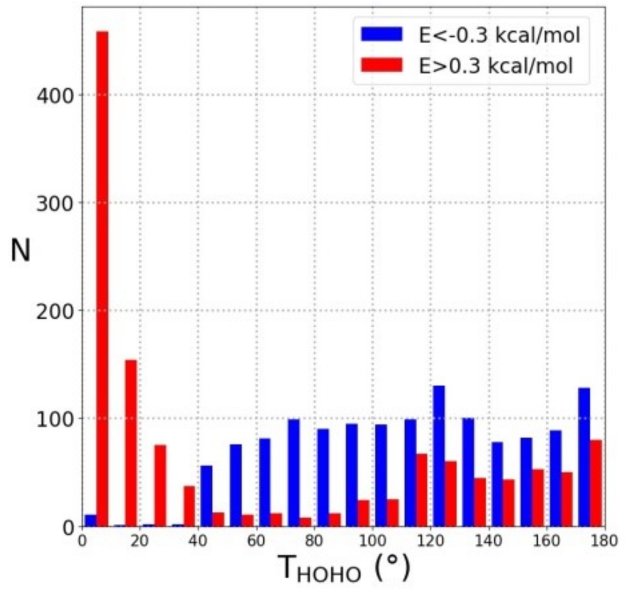


**Figure S4** The distribution of attractive (blue) and repulsive (red) interaction energies for various torsion angle $\boldsymbol{T_{HOHO}}$ (**Figure 2**) values, of antiparallel interactions found in CSD after applying criteria: $\boldsymbol{\beta_1}, \boldsymbol{\beta_2} \geq 160°$. The interaction energies were calculated at CCSD(T)/CBS level of theory.

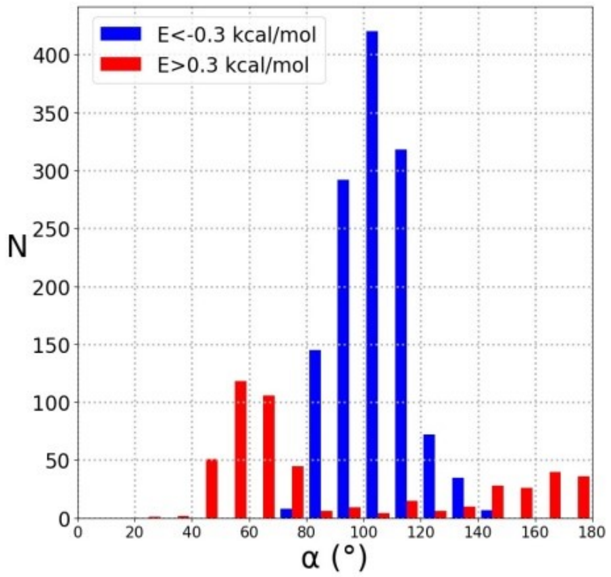


**Figure S5** The distribution of attractive (blue) and repulsive (red) interaction energies for various angle $\boldsymbol{\alpha}$ (**Figure** 2) values, of antiparallel interactions found in CSD after applying criteria: $\boldsymbol{\beta_1}$, $\boldsymbol{\beta_2} \geq 160°$ and $\boldsymbol{T_{HOHO}} > 40°$. The interaction energies were calculated at CCSD(T)/CBS level of theory.

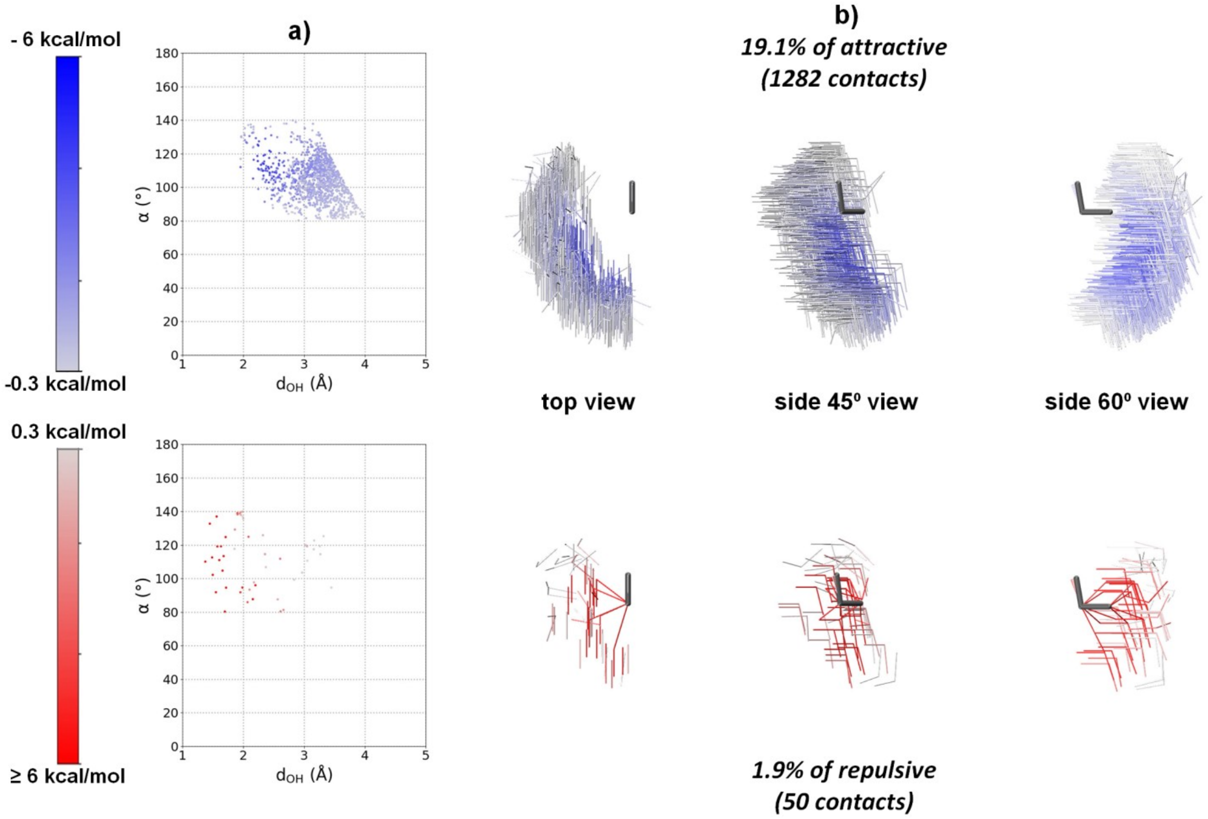


**Figure S6** Water-water contacts in the CSD structures. Colors correspond to interaction energies, blue (attractive) and red (repulsive), as was shown by the scale. *a)* The plots of the distance $d_{OH}$ (**Figure 2**) versus the angle $\alpha$ (**Figure 2**) for attractive (1282) and repulsive (50) contacts of antiparallel interactions found in CSD after applying criteria: $\beta_1, \beta_2 \geq 160°$, $80° \leq \alpha \leq 140°$ and $T_{HOHO} > 40°$ (**Figure 4**). *b)* Graphical representations of water-water contacts corresponding to *a*. In the graphical representations of water-water contacts, one water molecule was positioned in the center (shown in dark gray licorice) and the other water molecule from every water-water contact is shown in the color representing the energy of the interaction.

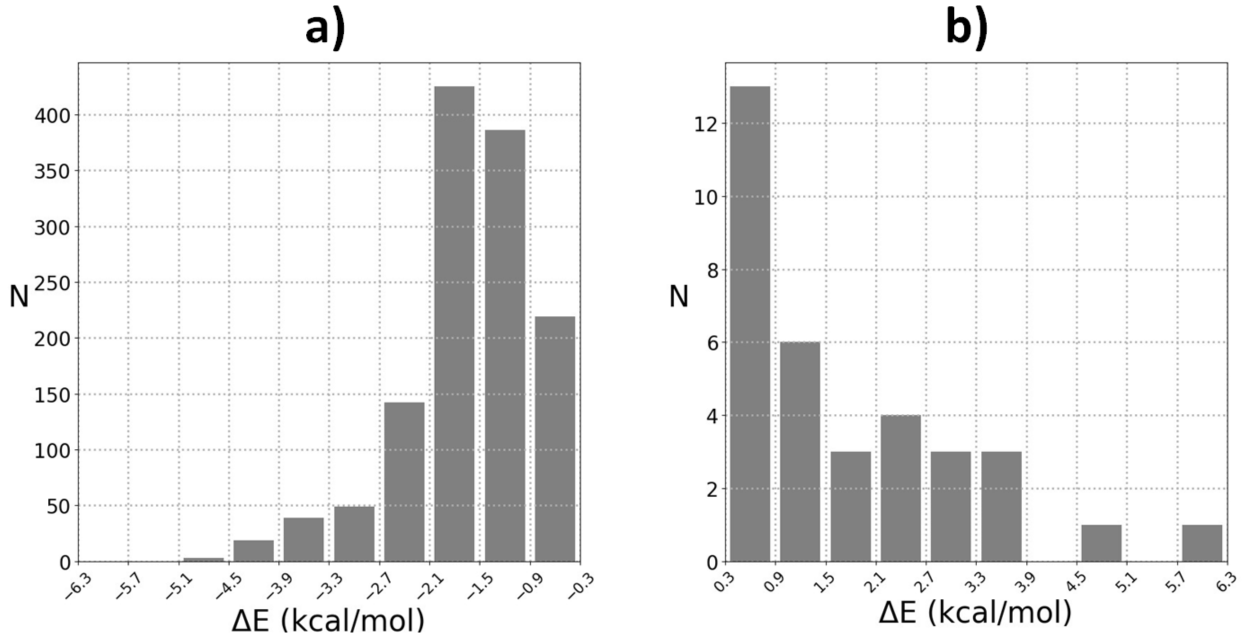


**Figure S7** The distribution of interaction energies of antiparallel interactions found in CSD after applying criteria: $\boldsymbol{\beta_1}$, $\boldsymbol{\beta_2} \geq 160°$, $80° \leq \boldsymbol{\alpha} \leq 140°$ and $\boldsymbol{T_{HOHO}} > 40°$ (**Figure 4**), *a)* 1282 and *b)* 50 repulsive contacts, calculated at CCSD(T)/CBS level of theory. The distribution corresponds to the structures shown in **Figure S4**.

**Table S1** Results of the SAPT analysis on some of the geometries of water-water contacts belonging to the group of antiparallel interactions (***$\beta_1$***, ***$\beta_2$*** ≥ 160°, 80° ≤ ***$\alpha$*** ≤ 140° and ***$T_{HOHO}$*** > 40°, **Figure 4**).

| Refcode | CCSD(T)/CBS[a] | Electrostatics[a] | Exchange[a] | Induction[a] | Dispersion[a] | Net Dispersion[a] | Total SAPT2+3[a] |
|---|---|---|---|---|---|---|---|
| KEFTUN | -3.50 | -6.00 | 5.64 | -0.98 | -2.13 | 3.51 | -3.47 |
| OFUDAW | -2.51 | -2.18 | 0.39 | -0.16 | -0.55 | -0.17 | -2.51 |
| AJIXOG | -2.50 | -2.19 | 0.70 | -0.21 | -0.77 | -0.08 | -2.48 |
| HEGTOE | -1.51 | -1.62 | 1.00 | -0.16 | -0.75 | 0.25 | -1.53 |
| RAXDEA | -1.50 | -1.74 | 1.58 | -0.26 | -1.02 | 0.56 | -1.44 |
| ECUYEJ | -1.49 | -1.85 | 1.45 | -0.20 | -0.92 | 0.53 | -1.52 |
| SOGCEX | -1.49 | -1.39 | 0.53 | -0.10 | -0.55 | -0.02 | -1.51 |
| BATFAD | -1.00 | -0.81 | 0.96 | -0.23 | -0.83 | 0.13 | -0.91 |
| HELLIW | -0.97 | -2.24 | 2.88 | -0.33 | -1.34 | 1.54 | -1.03 |
| OFOGOH01 | -0.50 | -0.23 | 0.10 | -0.07 | -0.28 | -0.18 | -0.48 |
| HADDUM | -0.49 | -4.45 | 9.99 | -2.60 | -3.51 | 6.48 | -0.56 |
| UCUQOZ | -0.49 | -0.46 | 1.13 | -0.18 | -0.90 | 0.23 | -0.42 |
| WIKSUH | -0.48 | -0.33 | 0.21 | -0.06 | -0.30 | -0.09 | -0.49 |

[a] All energies are in kcal/mol

**Table S2** Geometric parameters (**Figure 2**) of selected geometries (**Tables 1** and **S1**) of water-water contacts belonging to the group of antiparallel interactions ($\boldsymbol{\beta_1}, \boldsymbol{\beta_2} \geq 160°$, $80° \leq \boldsymbol{\alpha} \leq 140°$ and $\boldsymbol{T_{HOHO}} > 40°$, **Figure 4**).

| Refcode | $\Delta E$ [kcal/mol] | $d_{OO}$ [Å] | $d_{OH}$ [Å] | $d_{HH}$ [Å] | $\alpha$ [º] | $P_a/P_b$ [º] | $T_{HOHO}$ [º] | $\beta_1$ [º] | $B_2$ [º] | Ref. |
|---|---|---|---|---|---|---|---|---|---|---|
| IGOLEX | -4.65 | 2.885 | 2.336 | 2.136 | 113.91 | 0.00 | 127.41 | 180.00 | 70.25 | 1 |
| QICTAY | -4.04 | 2.845 | 2.232 | 1.961 | 118.62 | 0.00 | 102.88 | 180.00 | 72.68 | 2 |
| GITHUP | -3.52 | 2.906 | 2.575 | 2.605 | 99.35 | 0.00 | 164.06 | 180.00 | 70.47 | 3 |
| KEFTUN | -3.50 | 2.91 | 2.433 | 2.313 | 108.82 | 0.00 | 103.25 | 180.00 | 70.56 | 4 |
| CARNEO | -3.03 | 2.984 | 2.68 | 2.727 | 97.91 | 0.00 | 173.86 | 180.00 | 74.57 | 5 |
| XECJEU01 | -3.01 | 3.294 | 2.79 | 2.587 | 112.03 | 0.00 | 174.88 | 180.00 | 69.99 | 6 |
| OFUDAW | -2.51 | 3.415 | 3.015 | 2.913 | 105.39 | 0.00 | 153.12 | 180.00 | 69.31 | 7 |
| AJIXOG | -2.50 | 3.166 | 2.858 | 2.879 | 98.79 | 0.00 | 179.77 | 180.00 | 80.30 | 8 |
| HUBGAO | -2.00 | 3.249 | 3.024 | 3.115 | 94.07 | 0.00 | 151.90 | 180.00 | 69.98 | 9 |
| TEQKOQ | -2.00 | 3.781 | 3.13 | 2.696 | 124.41 | 0.00 | 101.40 | 180.00 | 69.58 | 10 |
| KUXTAZ | -1.98 | 3.132 | 2.798 | 2.772 | 100.33 | 0.00 | 76.02 | 80.16 | 180.00 | 11 |
| AQMLCO | -1.72 | 3.167 | 2.884 | 2.923 | 97.34 | 0.00 | 80.47 | 77.36 | 180.00 | 12 |
| HEGTOE | -1.51 | 3.414 | 3.135 | 3.158 | 97.77 | 0.00 | 80.16 | 180.00 | 74.33 | 13 |
| RAXDEA | -1.50 | 3.389 | 2.843 | 2.578 | 115.41 | 0.00 | 62.96 | 180.00 | 67.43 | 14 |
| ECUYEJ | -1.49 | 3.293 | 3.058 | 3.08 | 94.82 | 0.00 | 80.54 | 76.39 | 180.00 | 15 |
| SOGCEX | -1.49 | 3.634 | 3.334 | 3.17 | 99.54 | 0.00 | 69.49 | 79.74 | 180.00 | 16 |
| BATFAD | -1.00 | 3.048 | 3.032 | 3.326 | 81.55 | 0.00 | 179.36 | 180.00 | 72.49 | 17 |
| PASPOR | -1.00 | 3.499 | 3.368 | 3.307 | 89.26 | 0.00 | 77.65 | 72.15 | 180.00 | 18 |
| OCIDAI | -1.00 | 3.932 | 3.67 | 3.668 | 97.90 | 0.00 | 88.94 | 180.00 | 72.43 | 19 |
| HELLIW | -0.97 | 3.054 | 2.942 | 3.013 | 86.91 | 0.00 | 84.81 | 81.59 | 180.00 | 20 |
| OFOGOH01 | -0.50 | 3.656 | 3.627 | 3.862 | 83.82 | 0.00 | 177.48 | 180.00 | 68.79 | 21 |
| HADDUM | -0.49 | 2.782 | 2.017 | 1.539 | 132.12 | 0.00 | 174.93 | 180.00 | 68.89 | 22 |
| UCUQOZ | -0.49 | 3.503 | 3.033 | 2.638 | 110.23 | 0.00 | 45.82 | 74.33 | 180.00 | 23 |
| WIKSUH | -0.48 | 3.806 | 3.771 | 3.955 | 84.47 | 0.00 | 95.17 | 73.11 | 180.00 | 24 |

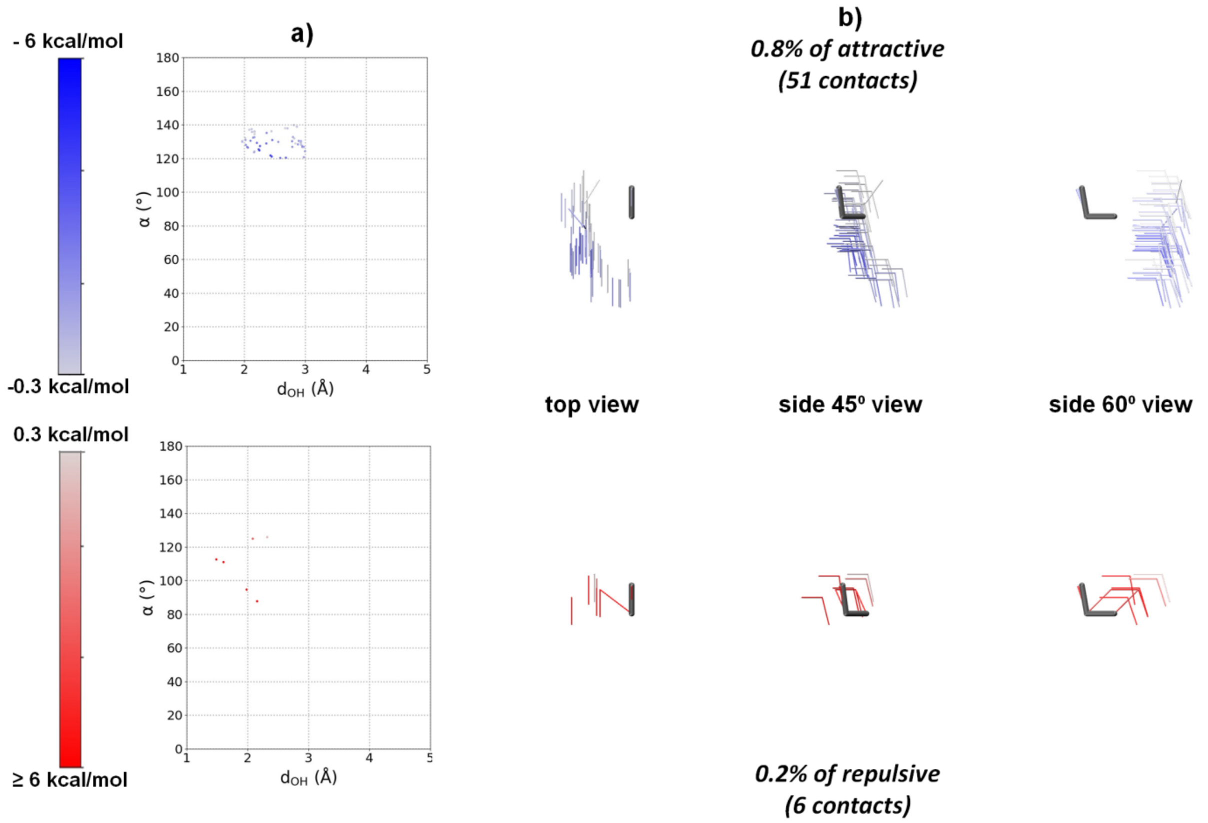


**Figure S8** Water-water contacts in the CSD structures. Colors correspond to interaction energies, blue (attractive) and red (repulsive), as was shown by the scale. *a)* The plots of the distance $\boldsymbol{d_{OH}}$ (**Figure 2**) versus the angle $\boldsymbol{\alpha}$ (**Figure 2**) for attractive (51) and repulsive (6) contacts of a small overlap of the two sets, the set of classical hydrogen bonds ($\boldsymbol{d_{OH}} \leq 3.0$ Å and $\boldsymbol{\alpha} \geq 120°$, **Figure 4**) and the set of antiparallel interactions ($\boldsymbol{\beta_1}$, $\boldsymbol{\beta_2} \geq 160°$, $80° \leq \boldsymbol{\alpha} \leq 140°$ and $\boldsymbol{T_{HOHO}} > 40°$, **Figure 4**). *b)* Graphical representations of water-water contacts corresponding to *a*. Colors correspond to interaction energies, blue (attractive) and red (repulsive), as was shown by the scale. In the graphical representations of water-water contacts, one water molecule was positioned in the center (shown in dark gray licorice) and the other water molecule from every water-water contact is shown in the color representing the energy of the interaction.

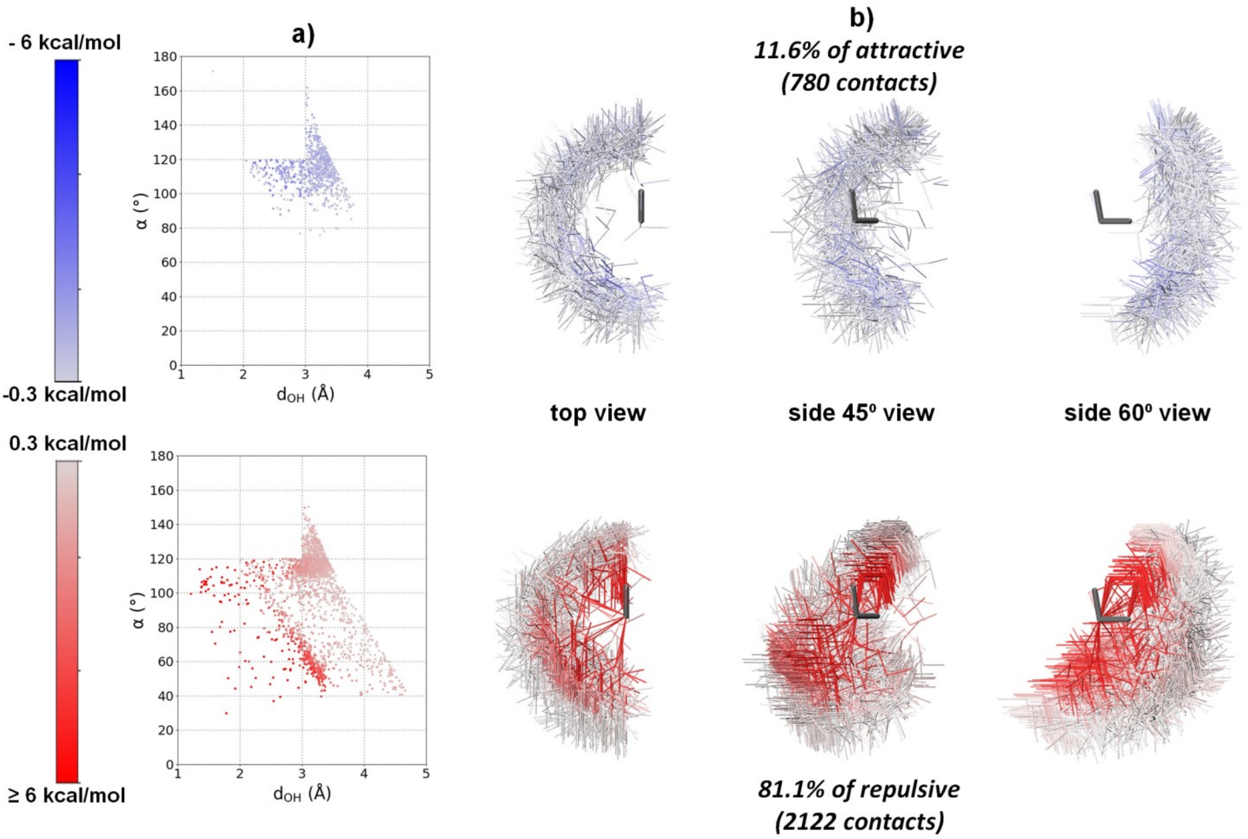


**Figure S9** Water-water contacts in the CSD structures. Colors correspond to interaction energies, blue (attractive) and red (repulsive), as was shown by the scale. *a)* The plots of the distance $\boldsymbol{d_{OH}}$ (**Figure 2**) versus the angle $\boldsymbol{\alpha}$ (**Figure 2**) for attractive (780) and repulsive (2122) contacts found in CSD remained after applying all criteria used to define groups: of majority classical hydrogen bonds ($\boldsymbol{d_{OH}} \leq$ 3.0 Å and $\boldsymbol{\alpha} \geq 120°$, **Figure 4**) and of antiparallel interactions ($\boldsymbol{\beta_1}$, $\boldsymbol{\beta_2} \geq 160°$, $80° \leq \boldsymbol{\alpha} \leq 140°$ and $\boldsymbol{T_{HOHO}}$ > 40°, **Figure 4**). *b)* Graphical representations of water-water contacts corresponding to *a*. In the graphical representations of water-water contacts, one water molecule was positioned in the center (shown in dark gray licorice) and the other water molecule from every water-water contact is shown in the color representing the energy of the interaction.

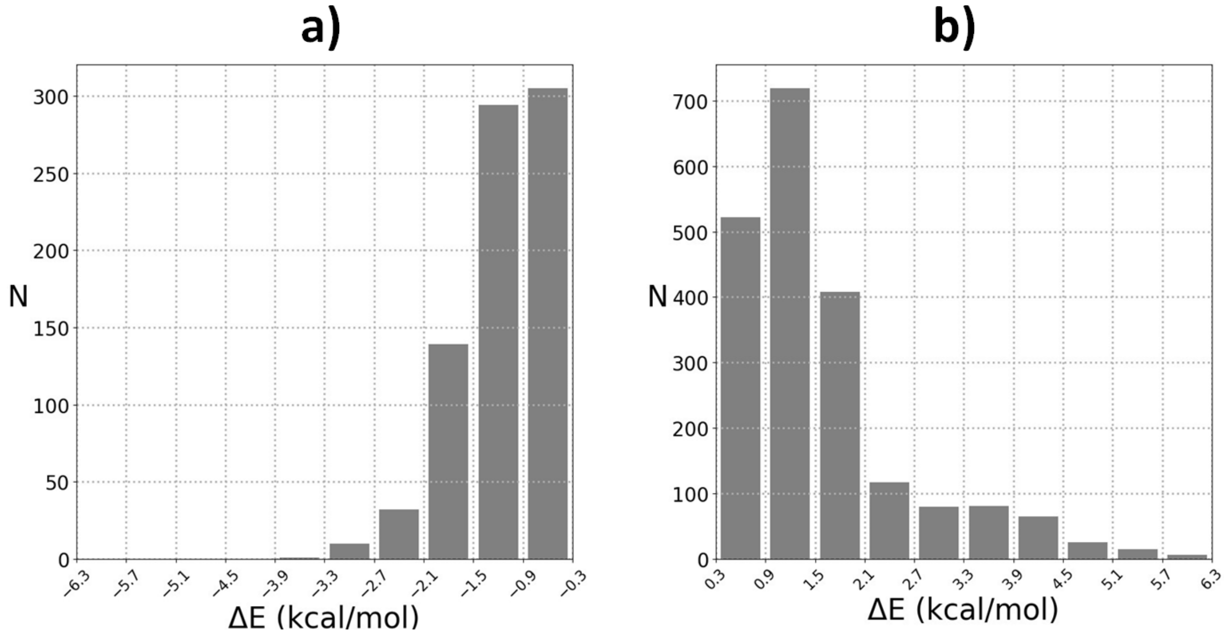


**Figure S10** The distribution of interaction energies of water-water contacts found in CSD remained after applying all criteria used to define groups: of majority classical hydrogen bonds ($d_{OH}$ ≤ 3.0 Å and $\alpha$ ≥ 120°, **Figure 4**) and of antiparallel interactions ($\beta_1$, $\beta_2$ ≥ 160°, 80° ≤ $\alpha$ ≤ 140° and $T_{HOHO}$ > 40°, **Figure 4**), *a)* 780 attractive and *b)* 2122 repulsive contacts, calculated at CCSD(T)/CBS level of theory. The distribution corresponds to the structures shown in **Figure S7**.

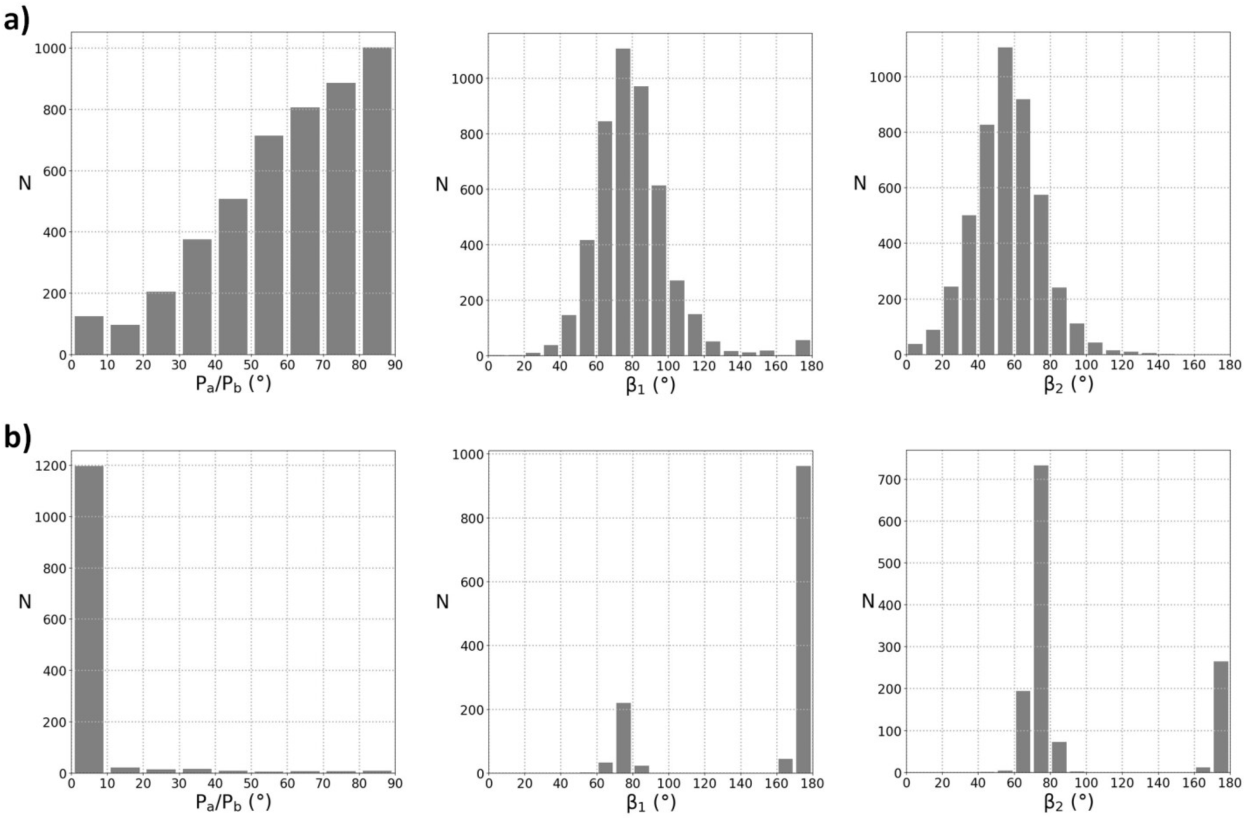

**Figure S11** Distributions of the angle $\boldsymbol{P_a/P_b}$ (**Figure 2**) and the angles between vectors $\boldsymbol{\beta_1}$, $\boldsymbol{\beta_2}$ (**Figure 2**) of *a)* the group of classical hydrogen bonds ($\boldsymbol{d_{OH}} \leq 3.0$ Å and $\boldsymbol{\alpha} \geq 120°$, **Figure 4**) and *b)* the group of antiparallel interactions ($\boldsymbol{\beta_1}$, $\boldsymbol{\beta_2} \geq 160°$, $80° \leq \boldsymbol{\alpha} \leq 140°$ and $\boldsymbol{T_{HOHO}} > 40°$, **Figure 4**).

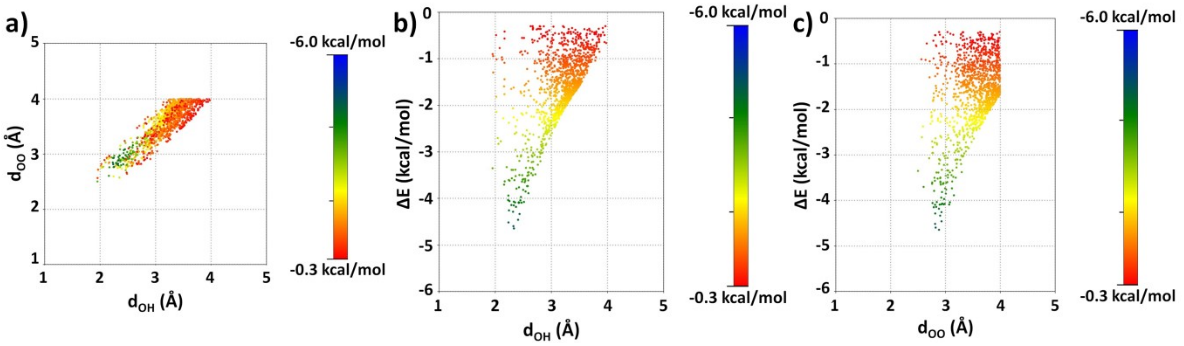

**Figure S12** Attractive water-water contacts in the CSD structures. Colors correspond to CCSD(T)/CBS interaction energies, as shown in the scale. *a)* The plot of the distance $\boldsymbol{d_{OH}}$ (**Figure 2**) versus the distance $\boldsymbol{d_{OO}}$ (**Figure 2**); *b)* the plot of the distance $\boldsymbol{d_{OH}}$ (**Figure 2**) versus the interaction energies; *c)* the plot of the distance $\boldsymbol{d_{OO}}$ (**Figure 2**) versus the interaction energies for attractive (1282) antiparallel interactions found in CSD after applying criteria: $\boldsymbol{\beta_1}$, $\boldsymbol{\beta_2} \geq 160°$, $80° \leq \boldsymbol{\alpha} \leq 140°$ and $\boldsymbol{T_{HOHO}} > 40°$ (**Figure 4**).

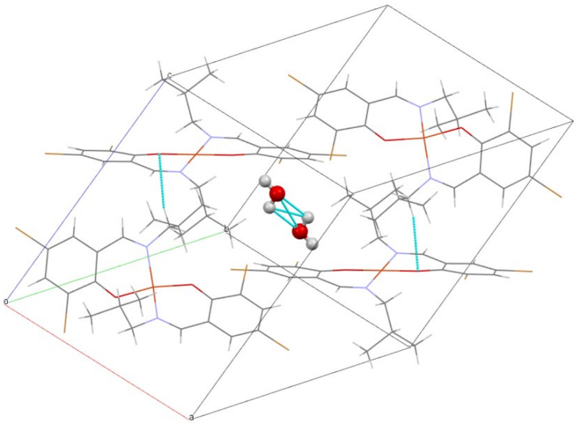

**Figure S13** A part of crystal structure KEJCOT representing antiparallel water-water interactions. All short contacts occurring in are shown with blue lines.

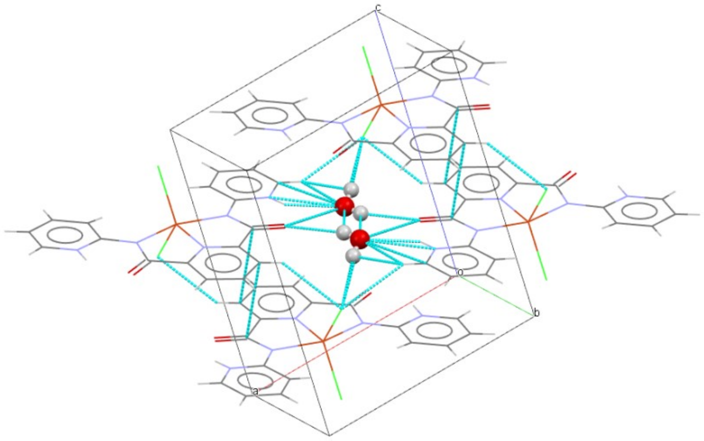

**Figure S14** A part of crystal structure TASZET representing antiparallel water-water interactions. All short contacts occurring in are shown with blue lines.

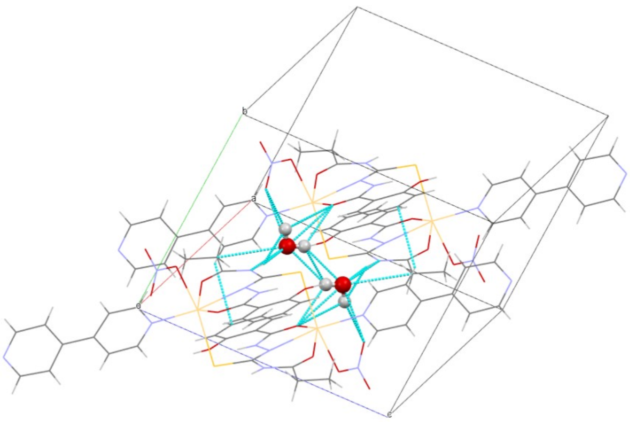

**Figure S15** A part of crystal structure XAFVAE representing antiparallel water-water interactions. All short contacts occurring in are shown with blue lines.

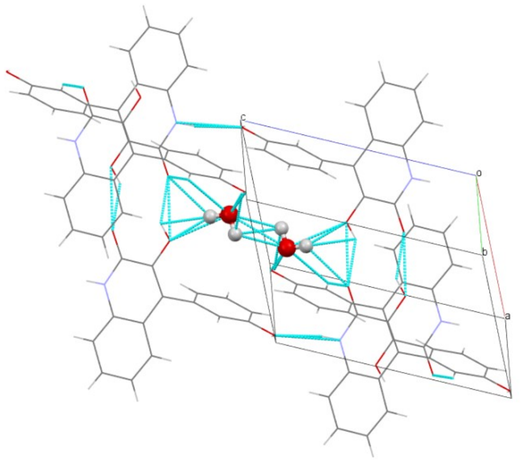

**Figure S16** A part of crystal structure EVOLUX representing antiparallel water-water interactions. All short contacts occurring in are shown with blue lines.

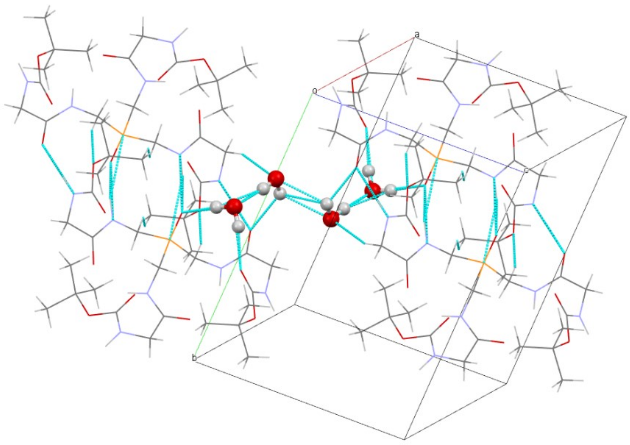

**Figure S17** A part of crystal structure UVETIZ representing antiparallel water-water interactions. All short contacts occurring in are shown with blue lines.

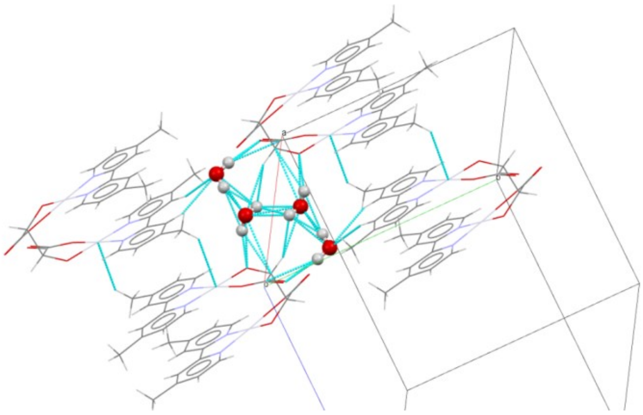

**Figure S18** A part of crystal structure MIKWIP representing antiparallel water-water interactions. All short contacts occurring in are shown with blue lines.

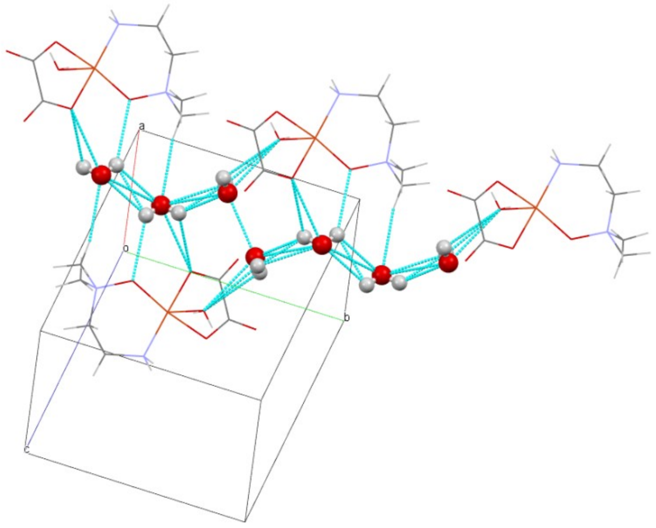


**Figure S19** A part of crystal structure AQOXCU representing antiparallel water-water interactions. All short contacts occurring in are shown with blue lines.

**Table S3** Geometric parameters (**Figure 2**) of water-water contacts in selected CSD crystal structures (**Figures S13-S19**).

| Refcode | Type[a] | $\Delta E$ [kcal/mol] | $d_{OO}$ [Å] | $d_{OH}$ [Å] | $d_{HH}$ [Å] | $\alpha$ [º] | $P_a/P_b$ [º] | $T_{HOHO}$ [º] | $\beta_1$ [º] | $B_2$ [º] | Ref. |
|---|---|---|---|---|---|---|---|---|---|---|---|
| KEJCOT | API | -4.60 | 2.824 | 2.327 | 2.198 | 109.86 | 0.00 | 149.78 | 180.00 | 70.61 | 25 |
| TASZET | API | -3.74 | 3.121 | 2.583 | 2.361 | 113.96 | 0.00 | 140.65 | 180.00 | 77.81 | 26 |
| XAFVAE | API | -4.04 | 3.084 | 2.451 | 2.117 | 121.10 | 0.00 | 119.82 | 180.00 | 75.46 | 27 |
| EVOLUX | API | -4.29 | 2.816 | 2.32 | 2.193 | 109.80 | 0.00 | 131.65 | 180.00 | 76.61 | 28 |
| UVETIZ | CHB | -4.43 | 2.826 | 1.841 | 2.348 | 171.18 | 87.60 | 84.46 | 80.55 | 59.06 | 29 |
| UVETIZ | API | -3.45 | 3.113 | 2.587 | 2.381 | 113.07 | 0.00 | 99.46 | 180.00 | 75.50 | 29 |
| MIKWIP | CHB | -2.09 | 2.773 | 1.79 | 1.843 | 169.98 | 81.73 | 16.21 | 112.84 | 66.61 | 30 |
| MIKWIP | API | -3.61 | 2.761 | 2.244 | 2.102 | 111.10 | 0.00 | 103.59 | 180.00 | 70.12 | 30 |
| AQOXCU | CHB | -2.93 | 2.858 | 2.075 | 2.021 | 134.24 | 76.07 | 121.36 | 124.96 | 103.55 | 31 |
| AQOXCU | API | -4.15 | 2.789 | 2.333 | 2.254 | 106.92 | 0.00 | 136.76 | 180.00 | 76.39 | 31 |

[a] API = antiparallel interaction (***$\beta_1$***, ***$\beta_2$*** ≥ 160°, 80° ≤ ***$\alpha$*** ≤ 140° and ***$T_{HOHO}$*** > 40°, **Figure 4**); CHB = classical hydrogen bond (***$d_{OH}$*** ≤ 3.0 Å and ***$\alpha$*** ≥ 120°, **Figure 4**)

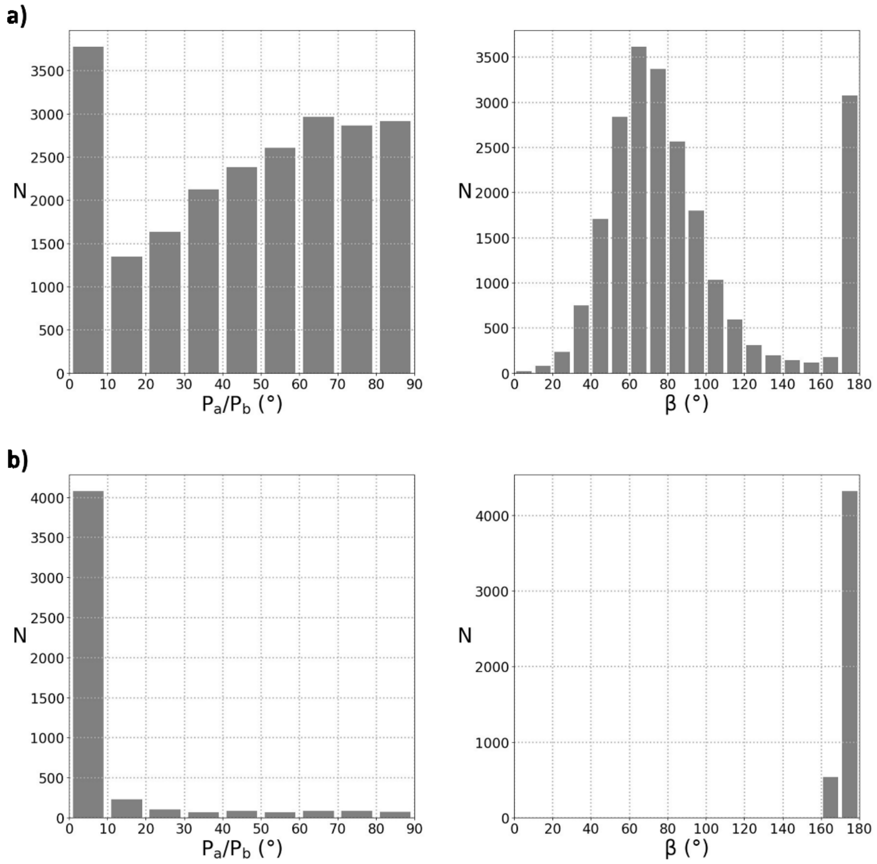


**Figure S20** Distributions of the angle $\boldsymbol{P_a/P_b}$ (**Figure 2**) and the angles between vectors $\boldsymbol{\beta}$ (**Figure 2**) for fragments containing O-H bond, majority of these structures (ca. 90.4%) are alcohols. *a)* The group of classical hydrogen bonded molecular ($\boldsymbol{d_{OH}} \leq 3.0$ Å and $\boldsymbol{\alpha} \geq 120°$) and *b)* the group of antiparallel oriented O-H bonds ($\boldsymbol{\beta} \geq 160°$, $80° \leq \boldsymbol{\alpha} \leq 140°$ and $\boldsymbol{T_{HOHO}} > 40°$). The data were obtained by following the procedure shown in **Figure 4**.

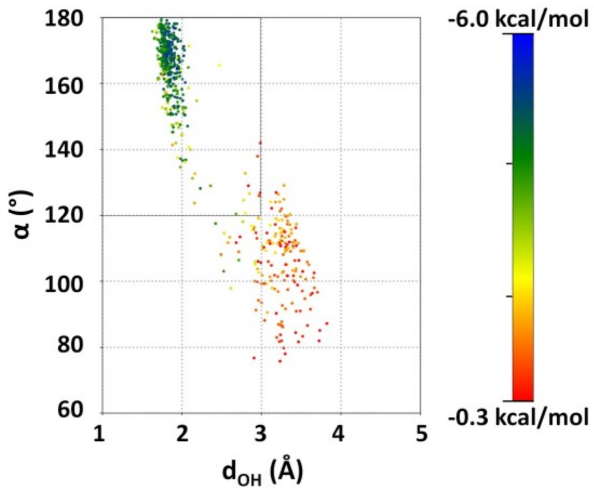


**Figure S21** Attractive water-water contacts in the CSD structures in which hydrogen atom positions were solved by difference Fourier map method. The plot of the distance $\boldsymbol{d_{OH}}$ versus the angle $\boldsymbol{\alpha}$. Colors correspond to CCSD(T)/CBS interaction energies, as shown in the scale.

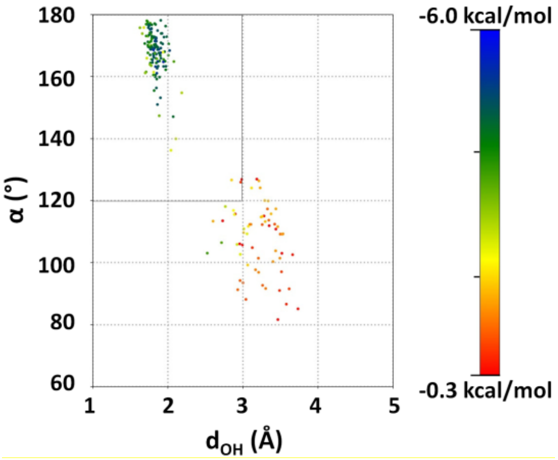


**Figure S22** Attractive water-water contacts in the CSD structures in which all atoms (including hydrogen atoms) were solved by difference Fourier map method and all hydrogen atom parameters were refined. The plot of the distance $d_{OH}$ versus the angle $\alpha$. Colors correspond to CCSD(T)/CBS interaction energies, as shown in the scale.

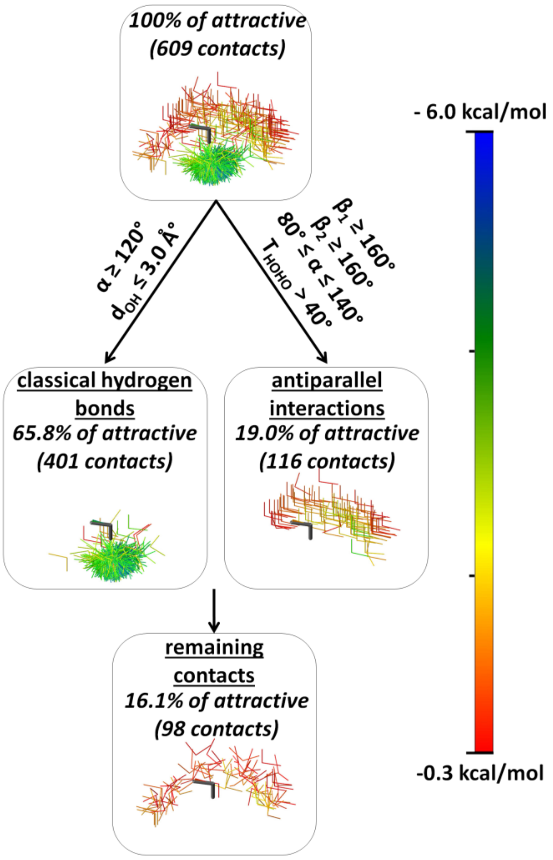


**Figure S23** Sets of attractive water-water contacts found in CSD in which hydrogen atom positions were solved by difference Fourier map method. In the graphical representations of water-water contacts, one water molecule was positioned in the center (shown in dark grey licorice). The other water molecules from every water-water contact are shown in the color representing the CCSD(T)/CBS interaction energies, as shown in the scale.

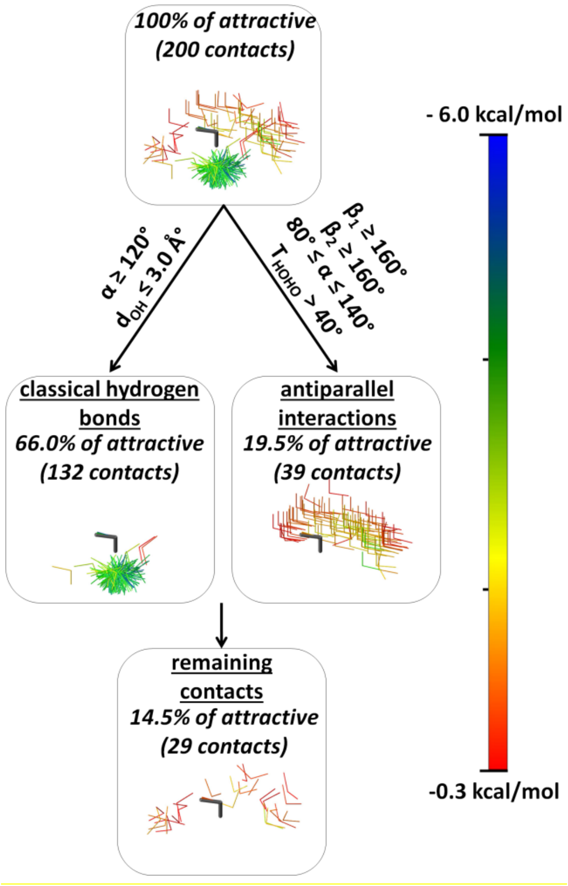


**Figure S24** Sets of attractive water-water contacts found in CSD in which all atoms (including hydrogen atoms) were solved by difference Fourier map method and all hydrogen atom parameters were refined. In the graphical representations of water-water contacts, one water molecule was positioned in the center (shown in dark grey licorice). The other water molecules from every water-water contact are shown in the color representing the CCSD(T)/CBS interaction energies, as shown in the scale.

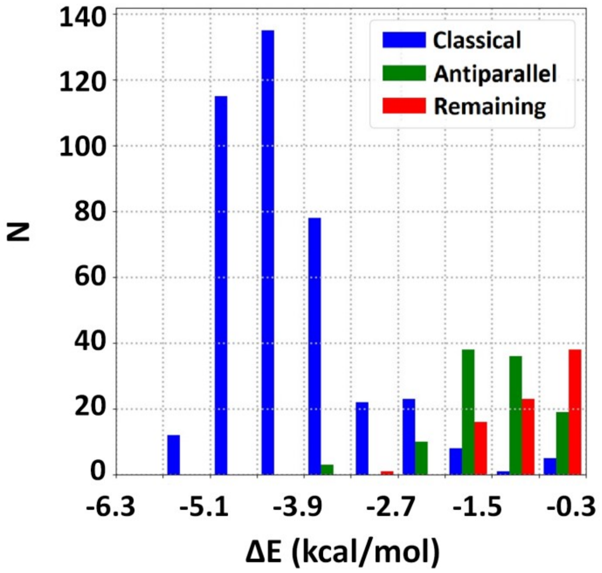


**Figure S25** The distribution of interaction energies of attractive water-water contacts found in CSD, in which hydrogen atom positions were solved by difference Fourier map method, after applying criteria for classical hydrogen bonds ($d_{OH} \leq 3.0$ Å and $\alpha \geq 120°$, **Figure S23**) and antiparallel interactions ($\beta_1$, $\beta_2 \geq 160°$, $80° \leq \alpha \leq 140°$ and $T_{HOHO} > 40$, **Figure S23**) calculated at CCSD(T)/CBS level of theory.

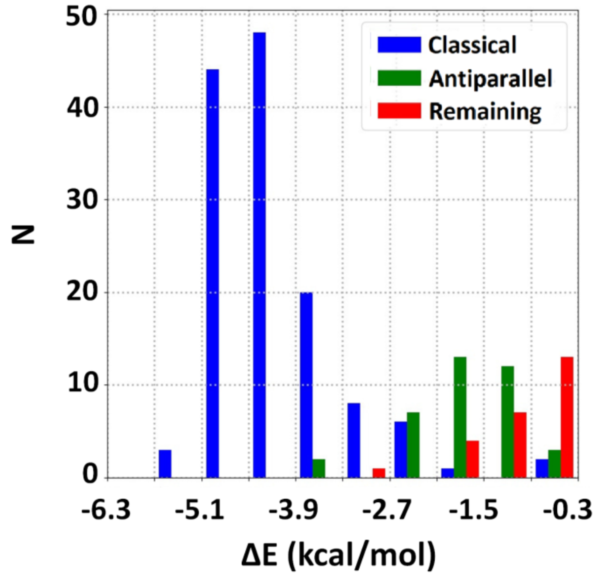


**Figure S26** The distribution of interaction energies of attractive water-water contacts found in CSD, in which all atoms (including hydrogen atoms) were solved by difference Fourier map method and all hydrogen atom parameters were refined, after applying criteria for classical hydrogen bonds ($d_{OH} \leq 3.0$ Å and $\alpha \geq 120°$, **Figure S24**) and antiparallel interactions ($\beta_1$, $\beta_2 \geq 160°$, $80° \leq \alpha \leq 140°$ and $T_{HOHO} > 40°$, **Figure S24**), calculated at CCSD(T)/CBS level of theory.

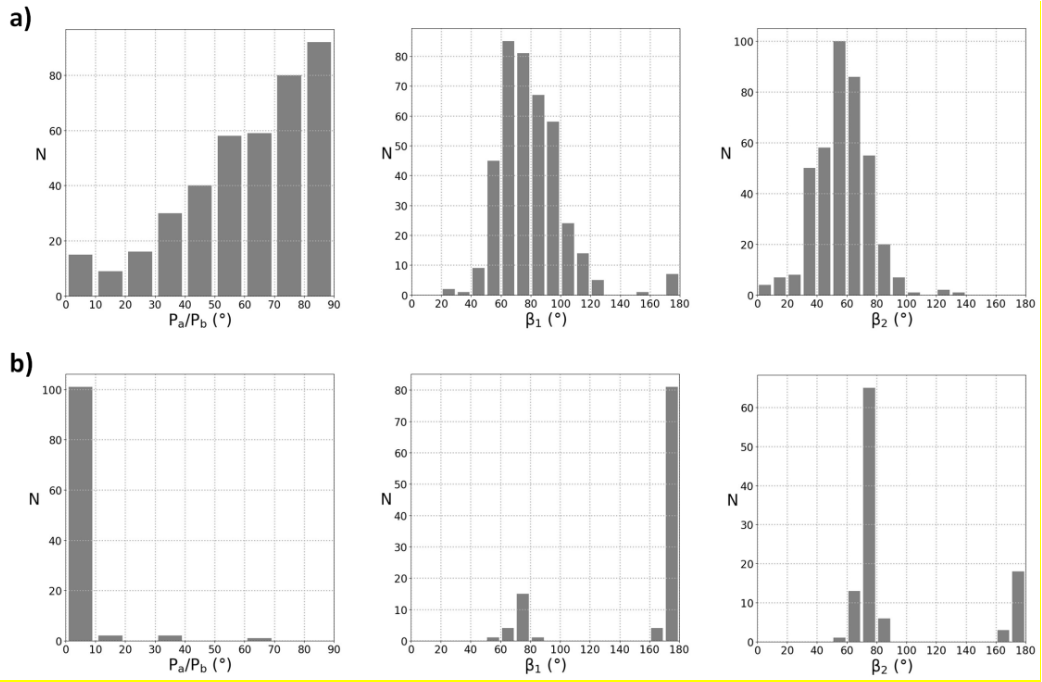


**Figure S27** Distributions of the angle $\boldsymbol{P_a/P_b}$ (**Figure 2**) and the angles between vectors $\boldsymbol{\beta_1}$, $\boldsymbol{\beta_2}$ (**Figure 2**) of *a)* the group of classical hydrogen bonds ($\boldsymbol{d_{OH}} \leq 3.0$ Å and $\boldsymbol{\alpha} \geq 120°$, **Figure S23**) and *b)* the group of antiparallel interactions ($\boldsymbol{\beta_1}$, $\boldsymbol{\beta_2} \geq 160°$, $80° \leq \boldsymbol{\alpha} \leq 140°$ and $\boldsymbol{T_{HOHO}} > 40°$, **Figure S23**), found in structures in which hydrogen atom positions were solved by difference Fourier map method.

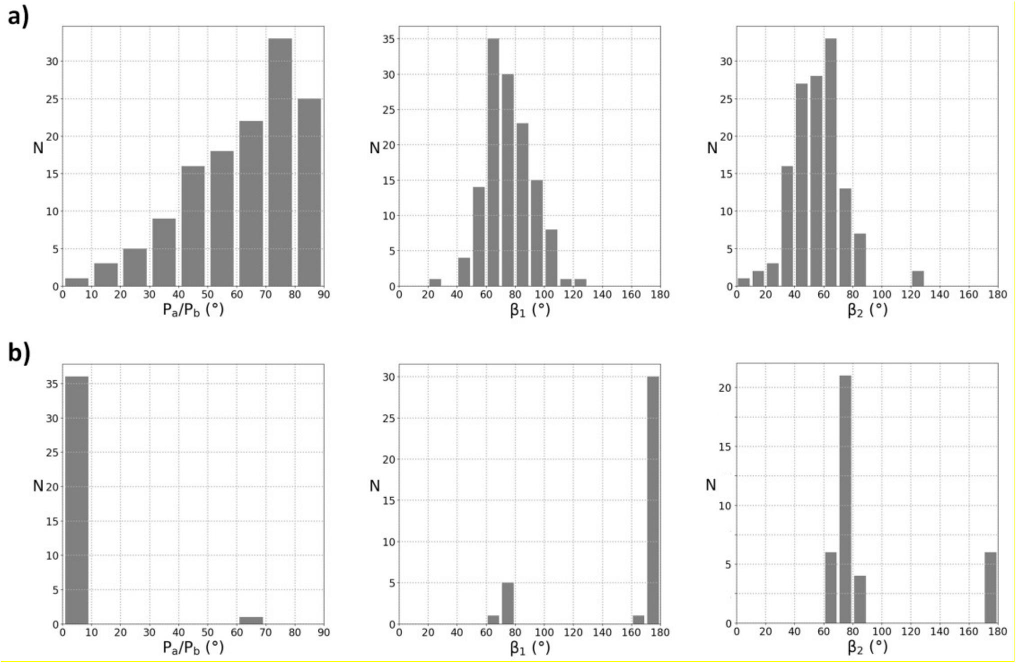


**Figure S28** Distributions of the angle $P_a/P_b$ (**Figure 2**) and the angles between vectors $\boldsymbol{\beta_1}$, $\boldsymbol{\beta_2}$ (**Figure 2**) of *a)* the group of classical hydrogen bonds ($\boldsymbol{d_{OH}} \leq 3.0$ Å and $\boldsymbol{\alpha} \geq 120°$, **Figure S24**) and *b)* the group of antiparallel interactions ($\boldsymbol{\beta_1}$, $\boldsymbol{\beta_2} \geq 160°$, $80° \leq \boldsymbol{\alpha} \leq 140°$ and $\boldsymbol{T_{HOHO}} > 40°$, **Figure S24**), found in structures in which all atoms (including hydrogen atoms) were solved by difference Fourier map method and all hydrogen atom parameters were refined.

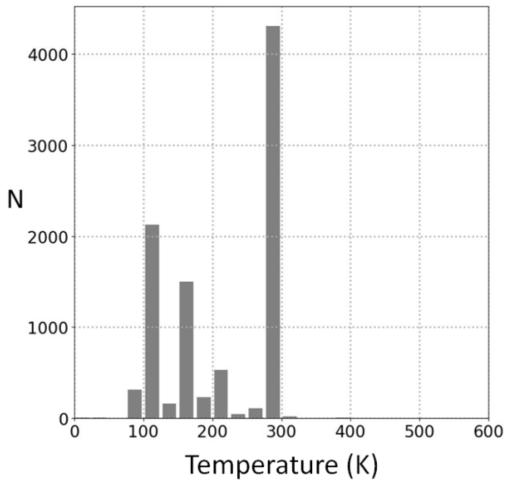


**Figure S29** The distribution of study temperatures that the considered crystal structures found in CSD were recorded at.

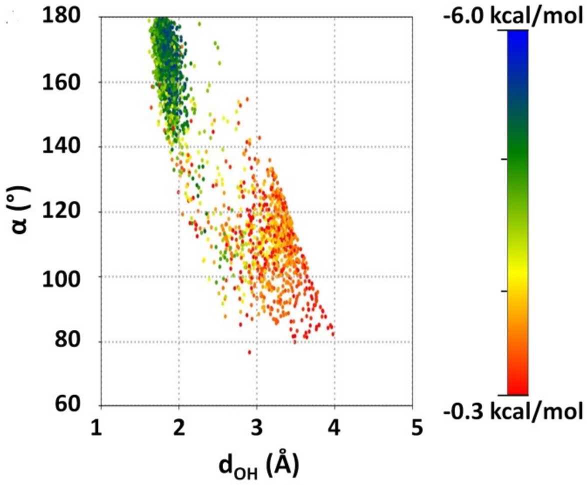


**Figure S30** Attractive water-water contacts in the CSD structures that are recorded at ≤ -78°C. The plot of the distance $d_{OH}$ versus the angle $\alpha$. Colors correspond to CCSD(T)/CBS interaction energies, as shown in the scale.

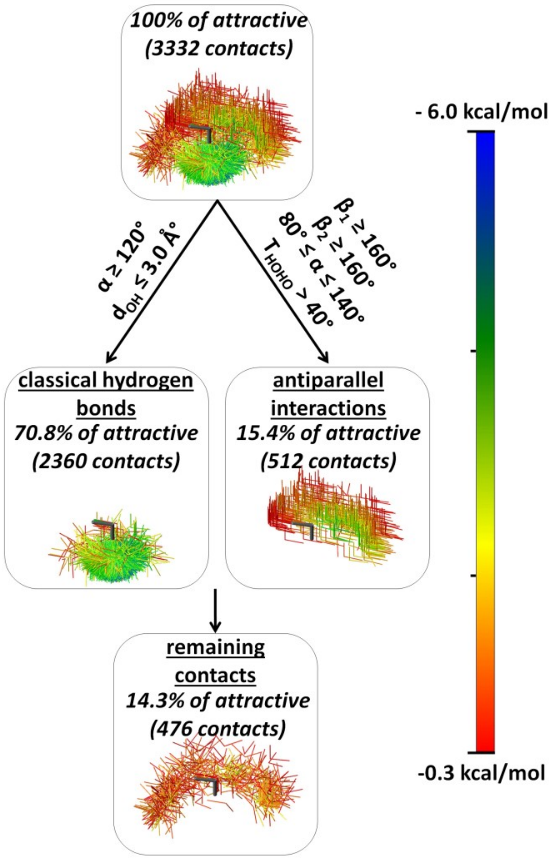


**Figure S31** Sets of attractive water-water contacts found in the CSD structures that are recorded at ≤ -78°C. In the graphical representations of water-water contacts, one water molecule was positioned in the center (shown in dark grey licorice). The other water molecules from every water-water contact are shown in the color representing the CCSD(T)/CBS interaction energies, as shown in the scale.

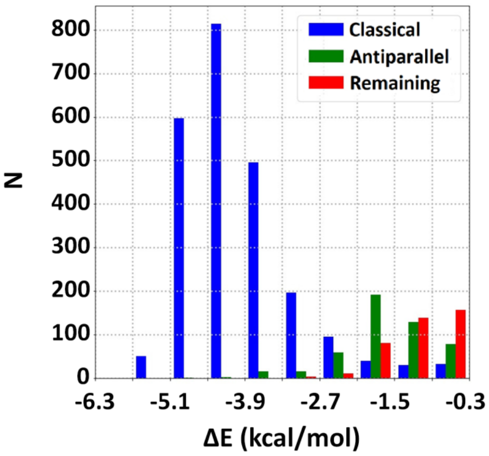


**Figure S32** The distribution of interaction energies of attractive water-water contacts found in the CSD that are recorded at ≤ -78°C, after applying criteria for classical hydrogen bonds ($d_{OH}$ ≤ 3.0 Å and $\alpha$ ≥ 120°, **Figure S24**) and antiparallel interactions ($\beta_1$, $\beta_2$ ≥ 160°, 80° ≤ $\alpha$ ≤ 140° and $T_{HOHO}$ > 40°, **Figure S24**), calculated at CCSD(T)/CBS level of theory.

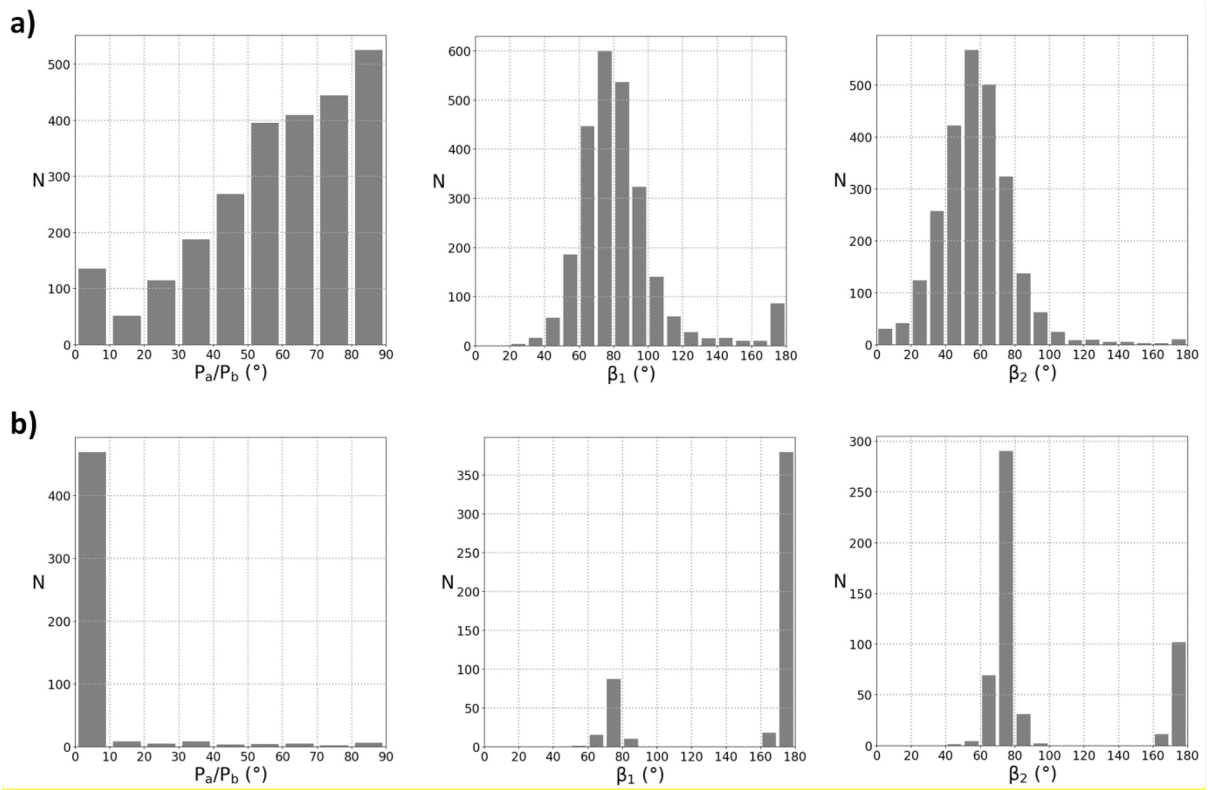


**Figure S33** Distributions of the angle $\boldsymbol{P_a/P_b}$ (**Figure 2**) and the angles between vectors $\boldsymbol{\beta_1}$, $\boldsymbol{\beta_2}$ (**Figure 2**) of *a)* the group of classical hydrogen bonds ($\boldsymbol{d_{OH}} \leq 3.0$ Å and $\boldsymbol{\alpha} \geq 120°$, **Figure S31**) and *b)* the group of antiparallel interactions ($\boldsymbol{\beta_1}$, $\boldsymbol{\beta_2} \geq 160°$, $80° \leq \boldsymbol{\alpha} \leq 140°$ and $\boldsymbol{T_{HOHO}} > 40°$, **Figure S31**), found in structures that are recorded at ≤ -78°C.

### S2. Details of overlapping and free web server

The aligned water molecules were those which contain hydrogen atom $H_{a1}$ (**Figure 2**). All the crystallographic translations were performed in order to account for the interactions between the original crystallographic cell and all its surrounding images. The hydrogen atoms of the aligned water molecules were distinguished by the proximity to the interacting oxygen atom $O_b$ (**Figure 2**). The oxygen atoms of the aligned water molecules were put at the same point, then the whole structure was transformed until the hydrogen atoms closer to the other water molecule ($H_{a1}$ atoms, **Figure 2**) are put along the same vector, and finally, the whole structure was transformed until the other hydrogen ($H_{a2}$, **Figure 2**) is put in the same plane. The water molecule symmetry ($C_{2v}$ group) was used to provide images that are easier to perceive. All the interacting water molecules were reflected to the same side of the aligned water plane (left side in figures).

As mentioned in the main text, given a .cif file and desired atom names of the interacting two water molecules as the input, the crystallographic structure is completed to the whole unit cell and then translated in all directions. The angles $\boldsymbol{\alpha}$ (**Figure 2**) of water molecules are checked for the range 96.4°

- 112.8º and the $\boldsymbol{d_{OO}}$ distance (**Figure 2**) is checked for condition shorter than 4.0 Å. The positions of hydrogen atoms are normalized so that the O-H bonds are 0.993 Å. All the scores that satisfy the criteria are given in the output. Namely, for the same cif structure and same atoms, there might be more than one water - water interaction differing in geometry, depending on the crystallographic transformations.

## S3. References

1. Zhou, H., Yu, D.-Y., Zhou, H.-B. & Yuan, A.-H. (2015). *Zeitschrift für anorganische und allgemeine Chemie* **641**, 858–862.

2. Rao, K. P. & Rao, C. N. R. (2007). *Inorg. Chem.* **46**, 2511–2518.

3. Ohui, K., Afanasenko, E., Bacher, F., Ting, R. L. X., Zafar, A., Blanco-Cabra, N., Torrents, E., Dömötör, O., May, N. V., Darvasiova, D., Enyedy, É. A., Popović-Bijelić, A., Reynisson, J., Rapta, P., Babak, M. V., Pastorin, G. & Arion, V. B. (2019). *J. Med. Chem.* **62**, 512–530.

4. Ghosh, S., Deka, H., Saha, S. & Mondal, B. (2017). *Inorg. Chim. Acta* **466**, 285–290.

5. Keana, J. F. W., Bland, J. S., Boyle, P. J., Erion, M., Hartling, R., Husman, J. R., Roman, R. B., Ferguson, G. & Parvez, M. (1983). *J. Org. Chem.* **48**, 3627–3631.

6. Vishweshwar, P., Nangia, A. & Lynch, V. M. (2002). *J. Org. Chem.* **67**, 556–565.

7. (a) Korotaev, V. Y., Barkov, Y., Slepukhin, P. A. & Sosnovskikh, V. Y. (2012). *Izvestiya Akademii Nauk SSSR, Seriya Khimicheskaya (Russ. Chem. Bull.)* 1734; (b) Korotaev, V. Y., Barkov, Y., Slepukhin, P. A. & Sosnovskikh, V. Y. (2013). *CCDC 863921: Experimental Crystal Structure Determination.*

8. Gilardi, R. & Evans, R. N. (2003). *Acta Cryst.* E**59**, o1226–o1227.

9. Lu, P., Yu, Y.-H., Chen, Z.-J., Hou, G.-F., Chen, Y.-M., Ma, D.-S., Gao, J.-S. & Gong, X.-F. (2015). *Synth. Met.* **203**, 164–173.

10. Zheng, Y.-Q., Xu, W., Lin, F. & Fang, G.-S. (2006). *J. Coord. Chem.* **59**, 1825–1834.

11. Ghosh, K., Sen, T., Fröhlich, R., Petsalakis, I. D. & Theodorakopoulos, G. (2010). *J. Phys. Chem. B* **114**, 321–329.

12. Karipides, A. (1981). *Acta Cryst.* B**37**, 1115–1117.

13. Zhu, H.-L., Xu, W., Wang, J.-F. & Zheng, Y.-Q. (2012). *Synth. Met.* **162**, 1327–1334.

14. (a) Sun, Y.-G., Gao, E.-J., Wei, D.-Z. & Liu, Y.-N. (2005). *Jiegou Huaxue (Chin. J. Struct. Chem.)* **24**, 1298; (b) Sun, Y.-G., Gao, E.-J., Wei, D.-Z. & Liu, Y.-N. (2006). *CCDC 219131: Experimental Crystal Structure Determination*.

15. Wang, R. & Seyedsayamdost, M. R. (2017). *Org. Lett.* **19**, 5138–5141.

16. Gao, Q., Gao, W.-H., Zhang, C.-Y. & Xie, Y.-B. (2008). *Acta Cryst.* E**64**, m928.

17. Klapötke, T. M., Krumm, B. & Polborn, K. (1999). *Eur. J. Inorg. Chem.* **1999**, 1359–1366.

18. Golovnev, N. N., Molokeev, M. S., Sterkhova, I. V., Lesnikov, M. K. & Atuchin, V. V., (2017). *Polyhedron* **134**, 120–125.

19. Tang, H., Guo, Y.-H., Sheng, J.-F., Tong, Y.-Z., Song, F., Wang, Z.-X., Qu, Z.-R. & Xuebao, W. H. (2017). *Chin. J. Inorg. Chem.* **33**, 134.

20. Ma, Q., Gu, H., Huang, J., Nie, F., Fan, G., Liao, L. & Yang, W. (2018). *New J. Chem.* **42**, 2376–2380.

21. Maiti, M., Sadhukhan, D., Thakurta, S., Zangrando, E., Pilet, G., Signorella, S., Bellú, S. & Mitra, S. (2014). *Bull. Chem. Soc. Jpn.* **87**, 724–732.

22. Hu, C.-J., Ren, X.-M., Lu, C.-S., Zou, Y., Zhang, W.-W., Duan, C.-Y., Meng, Q.-J. & Wang, H.-Q. (2003). *Transit. Met. Chem.* **28**, 350–355.

23. Sen, S., Choudhury, C. R., Talukder, P., Mitra, S., Westerhausen, M., Kneifel, A. N., Desplanches, C., Daro, N. & Sutter, J.-P. (2006). *Polyhedron* **25**, 1271–1278.

24. Al-Harthy, T. S., Shongwe, M. S., Husband, J., Stoll, R., Merz, K. & Abdel-Jalil, R. J. (2019). *J. Mol. Struc.* **1176**, 614–621.

1. Zhou, H., Yu, D.-Y., Zhou, H.-B. & Yuan, A.-H. (2015). *Zeitschrift für anorganische und allgemeine Chemie* **641**, 858–862.

2. Rao, K. P. & Rao, C. N. R. (2007). *Inorg. Chem.* **46**, 2511–2518.

3. Ohui, K., Afanasenko, E., Bacher, F., Ting, R. L. X., Zafar, A., Blanco-Cabra, N., Torrents, E., Dömötör, O., May, N. V., Darvasiova, D., Enyedy, É. A., Popović-Bijelić, A., Reynisson, J., Rapta, P., Babak, M. V., Pastorin, G. & Arion, V. B. (2019). *J. Med. Chem.* **62**, 512–530.

4. Ghosh, S., Deka, H., Saha, S. & Mondal, B. (2017). *Inorg. Chim. Acta* **466**, 285–290.

5. Keana, J. F. W., Bland, J. S., Boyle, P. J., Erion, M., Hartling, R., Husman, J. R., Roman, R. B., Ferguson, G. & Parvez, M. (1983). *J. Org. Chem.* **48**, 3627–3631.

6. Vishweshwar, P., Nangia, A. & Lynch, V. M. (2002). *J. Org. Chem.* **67**, 556–565.

7. (a) Korotaev, V. Y., Barkov, Y., Slepukhin, P. A. & Sosnovskikh, V. Y. (2012). *Izvestiya Akademii Nauk SSSR, Seriya Khimicheskaya (Russ. Chem. Bull.)* 1734; (b) Korotaev, V. Y., Barkov, Y., Slepukhin, P. A. & Sosnovskikh, V. Y. (2013). *CCDC 863921: Experimental Crystal Structure Determination.*

8. Gilardi, R. & Evans, R. N. (2003). *Acta Cryst.* E**59**, o1226–o1227.

9. Lu, P., Yu, Y.-H., Chen, Z.-J., Hou, G.-F., Chen, Y.-M., Ma, D.-S., Gao, J.-S. & Gong, X.-F. (2015). *Synth. Met.* **203**, 164–173.

10. Zheng, Y.-Q., Xu, W., Lin, F. & Fang, G.-S. (2006). *J. Coord. Chem.* **59**, 1825–1834.

11. Ghosh, K., Sen, T., Fröhlich, R., Petsalakis, I. D. & Theodorakopoulos, G. (2010). *J. Phys. Chem. B* **114**, 321–329.

12. Karipides, A. (1981). *Acta Cryst.* B**37**, 1115–1117.

13. Zhu, H.-L., Xu, W., Wang, J.-F. & Zheng, Y.-Q. (2012). *Synth. Met.* **162**, 1327–1334.

14. (a) Sun, Y.-G., Gao, E.-J., Wei, D.-Z. & Liu, Y.-N. (2005). *Jiegou Huaxue (Chin. J. Struct. Chem.)* **24**, 1298; (b) Sun, Y.-G., Gao, E.-J., Wei, D.-Z. & Liu, Y.-N. (2006). *CCDC 219131: Experimental Crystal Structure Determination*.

15. Wang, R. & Seyedsayamdost, M. R. (2017). *Org. Lett.* **19**, 5138–5141.

16. Gao, Q., Gao, W.-H., Zhang, C.-Y. & Xie, Y.-B. (2008). *Acta Cryst.* E**64**, m928.

17. Klapötke, T. M., Krumm, B. & Polborn, K. (1999). *Eur. J. Inorg. Chem.* **1999**, 1359–1366.

18. Golovnev, N. N., Molokeev, M. S., Sterkhova, I. V., Lesnikov, M. K. & Atuchin, V. V., (2017). *Polyhedron* **134**, 120–125.

19. Tang, H., Guo, Y.-H., Sheng, J.-F., Tong, Y.-Z., Song, F., Wang, Z.-X., Qu, Z.-R. & Xuebao, W. H. (2017). *Chin. J. Inorg. Chem.* **33**, 134.

20. Ma, Q., Gu, H., Huang, J., Nie, F., Fan, G., Liao, L. & Yang, W. (2018). *New J. Chem.* **42**, 2376–2380.

21. Maiti, M., Sadhukhan, D., Thakurta, S., Zangrando, E., Pilet, G., Signorella, S., Bellú, S. & Mitra, S. (2014). *Bull. Chem. Soc. Jpn.* **87**, 724–732.

22. Hu, C.-J., Ren, X.-M., Lu, C.-S., Zou, Y., Zhang, W.-W., Duan, C.-Y., Meng, Q.-J. & Wang, H.-Q. (2003). *Transit. Met. Chem.* **28**, 350–355.

23. Sen, S., Choudhury, C. R., Talukder, P., Mitra, S., Westerhausen, M., Kneifel, A. N., Desplanches, C., Daro, N. & Sutter, J.-P. (2006). *Polyhedron* **25**, 1271–1278.

24. Al-Harthy, T. S., Shongwe, M. S., Husband, J., Stoll, R., Merz, K. & Abdel-Jalil, R. J. (2019). *J. Mol. Struc.* **1176**, 614–621.

25. Cui, Y., Dong, X., Li, Y., Li, Z. & Chen, W. (2012). *Eur. J. Med. Chem.* **58**, 323–331.

26. Gudasi, K., Vadavi, R., Shenoy, R., Patil, M., Patil, S. A. & Nethaji, M. (2005). *Inorg. Chim. Acta.* **358**, 3799–3806.

27. Jiao, C., Zhang, S.-S., Li, Z.-Y., Liu, J.-J., Lin, M.-J. & Huang, C.-C. (2016). *Acta Cryst*. **C72**, 119–123.

28. Tao, Y.-W. & Wang, Y. (2011). *Acta Cryst.* **E67**, o2195–o2196.

29. Gavette, J. V., Lara, J., Berryman, O. B., Zakharov, L. N., Haley, M. M. & Johnson, D. W. (2011). *Chem. Commun*. **47**, 7653–7655.

30. Ohno, K., Kusano, Y., Kaizaki, S., Nagasawa, A. & Fujihara, T. (2018). *Inorg. Chem*. **57**, 14159–14169.

31. Pajunen, A. & Näsakkälä, M. (1980). *Acta Cryst*. **B36**, 1650–1651.
